# Cardiac MR Fingerprinting at 0.55T Using a Deep Image Prior for Joint T_1_ , T_2_ , and M_0_ Mapping

**DOI:** 10.1002/jmri.70239

**Published:** 2026-01-22

**Authors:** Zhongnan Liu, Zexuan Liu, Imran Rashid, Mauricio Stanzione Galizia, Christopher Scoma, William Truesdell, Prachi Agarwal, Nicole Seiberlich, Liyue Shen, Jesse Hamilton

**Affiliations:** ^1^ Department of Electrical Engineering and Computer Science University of Michigan Ann Arbor Michigan USA; ^2^ Department of Biomedical Engineering University of Michigan Ann Arbor Michigan USA; ^3^ School of Medicine Case Western Reserve University Cleveland Ohio USA; ^4^ Harrington Heart and Vascular Institute University Hospitals Cleveland Ohio USA; ^5^ Department of Radiology University of Michigan Ann Arbor Michigan USA; ^6^ Division of Cardiovascular Medicine University of Michigan Ann Arbor Michigan USA

**Keywords:** cardiac, deep learning, low field, MR fingerprinting, T_1_ mapping, T_2_ mapping

## Abstract

**Background:**

0.55T systems offer unique advantages and may support expanded access to cardiac MRI.

**Purpose:**

To assess the feasibility of 0.55T cardiac MR Fingerprinting (MRF), leveraging a deep image prior reconstruction to mitigate noise.

**Study Type:**

Phantom and prospective in vivo assessment.

**Population:**

ISMRM/NIST MRI system phantom and 18 healthy subjects (11 female; ages 28 ± 8 years).

**Field Strength and Sequences:**

MRF, modified Look‐Locker inversion recovery (MOLLI), and T_2_‐prepared balanced steady state free precession (T_2_‐bSSFP) at 0.55T.

**Assessment:**

MRF T_1_ and T_2_ maps were reconstructed using (1) a low‐rank technique with sparse and locally low‐rank regularization (SLLR‐MRF) and (2) a deep image prior (DIP‐MRF). Accuracy and precision of MRF and conventional sequences were evaluated in a phantom. In vivo performance of MRF was evaluated in the 18 healthy subjects, with 7 subjects also undergoing conventional mapping. Myocardial T_1_ and T_2_ values were compared among methods and image quality scored by three readers (2, 3, and 4 years of experience) on a 5‐point scale.

**Statistical Tests:**

Linear regression, Bland–Altman, intraclass correlation coefficient, and one‐way ANOVA with *p* < 0.05 considered significant.

**Results:**

Mean measurements in the left ventricular septum were 671 ± 31 ms (MOLLI), 761 ± 147 ms (SLLR‐MRF), and 686 ± 39 ms (DIP‐MRF) for T_1_, and 63.5 ± 5.7 ms (T_2_‐bSSFP), 47.5 ± 12.7 ms (SLLR‐MRF), and 45.2 ± 4.5 ms (DIP‐MRF) for T_2_. Compared to conventional mapping, DIP‐MRF exhibited significantly lower T_2_ but no differences in T_1_ (*p* > 0.99). Standard deviations within the myocardium were significantly lower with DIP‐MRF compared to SLLR‐MRF (39 vs. 147 ms for T_1_ and 4.5 vs. 12.7 ms for T_2_). Overall image quality ratings were significantly lower for SLLR‐MRF (T_1_: 2.3, T_2_: 2.9), which were significantly lower compared to conventional mapping methods (T_1_: 3.4, T_2_: 3.9), and DIP‐MRF (T_1_: 3.8, T_2_: 4.1) received higher scores.

**Data Conclusion:**

This study demonstrated the feasibility of cardiac MRF on a commercial 0.55T system, enabled by a deep image prior reconstruction for denoising.

**Evidence Level:**

2.

**Stage of Technical Efficacy:**

1.

## Plain Language Summary

While low‐field (0.55T) MRI scanners may decrease costs and facilitate improved access in low‐resource settings, they suffer from reduced signal that compromises image quality. This study introduces an approach to efficiently measure multiple clinically relevant properties in heart muscle at 0.55T by combining a rapid imaging framework—MR Fingerprinting (MRF)—with deep learning to attain high‐quality quantitative images. Feasibility is demonstrated in phantoms and 18 healthy subjects, where deep learning is shown to improve measurement precision, and MRF is rated by clinicians to have non‐inferior image quality compared to standard, less‐efficient techniques which measure these properties in separate scans.

## 1 Introduction

Quantitative cardiac MR enables non‐invasive characterization of myocardial tissue, with T_1_ and T_2_ mapping increasingly used to support the evaluation of various cardiac conditions [1]. T_1_ mapping aids in the assessment of myocardial inflammation, infarction, amyloidosis, lipid infiltration, and fibrosis [2, 3], while T_2_ mapping is effective for detecting myocardial edema, often associated with acute inflammatory or ischemic processes [4]. In clinical practice, cardiac T_1_ and T_2_ maps are typically acquired in separate 2D breathheld electrocardiogram (ECG)‐triggered scans. T_1_ mapping is commonly performed using modified Look‐Locker inversion recovery (MOLLI) [5] or saturation recovery single‐shot acquisition (SASHA) [6] sequences, while T_2_ maps are often acquired using a T_2_‐prepared balanced steady‐state free precession (T_2_‐bSSFP) sequence [7]. These techniques involve the acquisition of multiple T_1_‐ or T_2_‐weighted images, usually collected in a single‐shot manner with one image acquired per heartbeat, which are then fit to an exponential signal model to quantify relaxation times.

Despite their widespread use, these methods have several limitations. First, they are relatively inefficient, as mapping multiple tissue properties requires repeated breathholds that prolong the exam time. Second, maps can be misaligned due to motion occurring between separate T_1_ and T_2_ mapping scans, as well as misalignment between slices. Third, these methods are susceptible to various confounding factors, including heart rate variability, system imperfections, and tissue property interdependence, such as the influence of T_2_ relaxation on estimated T_1_ values and vice versa [8, 9].

Multiparametric techniques can address many of these limitations by measuring multiple properties within a single sequence. Several approaches have been developed for joint cardiac T_1_ and T_2_ mapping, including 3D‐QALAS [10], cardiac MR Multitasking [11], multiparametric SASHA [12], MultiMapping [13, 14], and MR fingerprinting (MRF) [15]. In particular, MRF performs multiparametric mapping using a time‐varying sequence that produces distinct magnetization signal evolutions (“fingerprints”) for different combinations of tissue property values. Data are collected using a highly undersampled (typically non‐Cartesian) acquisition, and maps are reconstructed by matching the measured fingerprint at each voxel to a dictionary of simulated fingerprints, where the best‐matching fingerprint determines the estimated tissue properties for a given voxel. Various implementations of MRF for cardiac imaging have been proposed, including the initial approach for joint T_1_ and T_2_ mapping [16], which has since been extended to quantify additional properties, including ventricular function (via cine MRF) [17], fat fraction [18, 19], T_1_ ρ [19, 20], and T_2_* [21].

Although cardiac MRI is traditionally performed at field strengths of 1.5 or 3 T, the emergence of commercial low‐field (e.g., 0.55T) scanners equipped with modern hardware has led to increased interest in extending and validating quantitative cardiac methods on these systems [22, 23]. Low‐field scanners offer several advantages for cardiac imaging, including reduced specific absorption rate, improved B_0_ and B_1_^+^ field homogeneity, decreased susceptibility artifacts (particularly near the heart‐lung interface and metal implants) [24], larger bore sizes to accommodate obese patients (a population with high prevalence of cardiovascular disease), and lower installation and operating costs (which could enable broader access to cardiac MRI). Several studies have demonstrated promising results at low field using both single‐parametric [23, 25] and multiparametric [19, 26] T_1_ and T_2_ mapping techniques.

However, a major challenge when performing quantitative mapping at 0.55T is the inherently lower signal‐to‐noise ratio (SNR), which can degrade map quality and precision [27]. One strategy to partially offset this limitation is to leverage the increased T_2_* relaxation times at 0.55T by using longer readouts that cover k‐space more efficiently. For example, Campbell‐Washburn et al. demonstrated that spirals provide more SNR‐efficient imaging compared to Cartesian sampling at 0.55T [23]. This makes MRF particularly well‐suited to low‐field imaging, as it commonly uses spiral trajectories. A complementary strategy is to use reconstruction methods to further suppress noise. While the original MRF work directly matched zero‐filled (undersampled) images to the dictionary, various model‐based [28, 29] and low‐rank reconstructions [30] for MRF have since been proposed. These methods often reduce noise via additional regularization terms, including total variation (TV) or locally low‐rank (LLR) constraints [31].

Deep learning has also emerged as a promising approach to reconstruct MRF data. While many deep learning methods require paired ground truth data for supervised training, acquiring such data is impractical for cardiac MRF, as a fully‐sampled acquisition would take several minutes, making it susceptible to cardiac and respiratory motion. Self‐supervised learning methods offer a more attractive solution, as they are trained directly on undersampled data without the need for extra training data, often combined with a forward physics model to ensure data consistency. One such technique previously applied to cardiac MRF at 1.5T is based on a deep image prior (DIP) approach [32]. DIP leverages the structure of a U‐Net as an implicit prior, with scan‐specific training performed on a single dataset to generate quantitative maps consistent with a low‐rank subspace model [33]. The U‐Net architecture preferentially captures low spatial frequency structures in the image before fitting to high‐frequency features (such as noise or aliasing artifacts), enabling high‐quality reconstructions when combined with early stopping or other forms of regularization to avoid overfitting [34]. The hypothesis of this study was that combining MRF with a DIP reconstruction could mitigate the inherently low SNR at 0.55T, thereby enabling simultaneous cardiac T_1_, T_2_, and proton density (M_0_) mapping during a single breathhold.

Thus, the aims of this study were to implement a 2D spiral fast imaging with steady state precession (FISP) cardiac MRF sequence on a commercial 0.55T system and compare the performance of an iterative low‐rank technique with sparse and locally low‐rank regularization (SLLR‐MRF) reconstruction and a DIP‐MRF reconstruction in both phantom and healthy subject scans. A further aim was to compare MRF image quality and quantitative measurements with those from conventional mapping sequences in healthy subjects.

## 2 Materials and Methods

### 2.1 Pulse Sequence

A 2D spiral cardiac MRF sequence originally developed for use at 1.5 and 3T [16] was adapted for a commercial 0.55T system (MAGNETOM Free.Max, Siemens Healthineers, Erlangen, Germany). A variable density spiral trajectory was designed to cover a 192 × 192 matrix over a 300 × 300 mm^2^ field of view (FOV), yielding an in‐plane resolution of 1.6 × 1.6 mm^2^. To accommodate the lower gradient performance of the 0.55T system, the spiral was designed with a maximum gradient amplitude of 17 mT/m, slew rate of 38 T/m/s, and readout duration of 6.3 ms. The spiral was rotated according to a pseudo golden angle scheme, where the desired rotation angle was calculated using the golden angle, and one of the 48 pre‐calculated spiral arms (equally spaced over 360°) was selected that was closest to this angle.

As in previous cardiac MRF implementations, the sequence employed a FISP readout with an unbalanced gradient moment along the slice‐selection axis, resulting in 8π dephasing across the voxel per TR [35]. Data were acquired during a 15‐heartbeat breathhold with prospective ECG triggering in a 243 ms diastolic acquisition window. Each diastolic window was preceded by a non‐selective preparation pulse that followed a five‐heartbeat pattern, repeated three times over the 15‐heartbeat scan. In the first heartbeat, an adiabatic inversion was applied followed by a 21 ms delay before data acquisition. No preparation was applied during the second heartbeat. In the third, fourth, and fifth heartbeats, a T_2_ preparation module with MLEV refocusing pulse [36] was applied with T_2_‐prep times of 30, 50, and 80 ms, respectively (Figure S1).

Within each acquisition period, data were collected using time‐varying flip angles between 4° and 25°. A key difference from higher‐field cardiac MRF implementations was the use of a longer TR of 9.0 ms (compared to 5.5 ms at 1.5 and 3T) due to the longer spiral readout. A total of 405 TRs were acquired during the scan, which is fewer than the 675–900 TRs collected in previous higher‐field cardiac MRF studies [16, 37]. This design choice was made to accommodate the reduced gradient performance of the 0.55T system without extending the breathhold or diastolic window.

### 2.2 Reconstruction

Given the inherently reduced SNR at 0.55T and shorter sequence length compared to previous cardiac MRF studies, two reconstruction methods were evaluated for image quality and quantitative mapping performance: (1) SLLR‐MRF, and (2) DIP‐MRF.

#### 2.2.1 Sparsity and Locally Low‐Rank MRF (SLLR‐MRF) Reconstruction

First, maps were reconstructed using an SLLR‐MRF technique [31]. A dictionary was generated using a Bloch equation simulation that incorporated scan‐specific cardiac rhythm timings from an ECG signal, as well as corrections for non‐ideal slice profile and preparation pulse efficiency [8]. The dictionary $D\in {\mathbb{C}}^{p\times t}$ contained *t* = 405 time points and approximately *p* = 23,000 fingerprints, with T_1_ values of 60–3000 ms and T_2_ values of 6–1000 ms, excluding combinations with T_2_ > T_1_. A truncated singular value decomposition (SVD) was performed to compress the dictionary along the temporal dimension, retaining only the top nine singular values that captured more than 99.99% of the signal energy [29]. The reconstruction was formulated as an inverse problem (Equation 1).

(1) $$\hat{x}=\arg\min_{x}\frac{1}{2}{\left\|\mathrm{FS}\left(xV_{k}^{*}\right)-y\right\|}_{2}^{2}+\sum_{b}\left(\lambda_{\mathrm{LLR}}{\left\|R_{b}x\right\|}_{*}\right)+\lambda_{\mathrm{TV}}{\left\|\nabla x\right\|}_{1}$$

The first term enforces data consistency, where x denotes the MRF subspace images, y is the acquired k‐space data, F represents the non‐uniform fast Fourier Transform (NUFFT) [38], and S denotes the coil sensitivity maps. $V_{k}\in {\mathbb{C}}^{t\times k}$ denotes the temporal basis functions (i.e., the first k columns of the right singular matrix) obtained from the SVD of the MRF dictionary. The second term imposes an LLR constraint via the nuclear norm ${\left\|\cdot \right\|}_{*}$, encouraging low‐rankness within local image patches, where the operator $R_{b}$ extracts spatially overlapping patches and reshapes them into Casorati matrices. The third term applies an $l_{1}$‐norm TV regularization, which promotes spatial sparsity and smoothness. The regularization weights $\lambda_{\mathrm{LLR}}$ and $\lambda_{\mathrm{TV}}$ control the balance between the data consistency and two regularization terms, respectively. This study used $\lambda_{\mathrm{LLR}}$ = 0.01 and $\lambda_{\mathrm{TV}}$ = 0.005 relative to the maximum signal intensity of the subspace images, selected empirically based on preliminary in vivo experiments (Figure S2). The reconstruction was solved using nonlinear conjugate gradient descent with 25 iterations. The resulting subspace images were then matched to the compressed dictionary to yield T_1_, T_2_ and M_0_ maps.

#### 2.2.2 Deep Image Prior MRF (DIP‐MRF) Reconstruction

Second, maps were reconstructed using DIP‐MRF, a self‐supervised deep learning technique that combines a low‐rank subspace model with a deep image prior framework [32]. DIP‐MRF performs self‐supervised training using undersampled k‐space data from a single scan, avoiding the need for extra reference training datasets for training the network. The reconstruction uses a system of untrained neural networks (Figure S3) to generate both MRF subspace images and quantitative maps without explicitly using a dictionary. An untrained U‐Net, referred to as the image reconstruction network (IRN) and denoted $\theta_{\mathrm{IRN}}$ is used to generate MRF subspace images (x) (Equation 2).

(2) $$x=\theta_{\mathrm{IRN}}\left(z\right)$$

The input $z\in {\mathbb{R}}^{n_{y}\times n_{x}\times d}$ is a fixed tensor initialized with uniform random numbers between −1 and 1, where $n_{y}$ and $n_{x}$ are the image dimensions and d is a tunable parameter defining the number of feature channels (set here to 32). The network output has size $n_{y}\times n_{x}\times 2k$, representing the real and imaginary parts of the subspace images. A second untrained network, termed the coil estimation network (CEN) and denoted $\theta_{\mathrm{CEN}}$, is used to estimate the coil sensitivity maps (Equation 3).

(3) $$S=\theta_{\mathrm{CEN}}\left(S_{0}\right)$$

This network takes an initial estimate of the coil sensitivities ($S_{0}$), derived from the time‐averaged MRF k‐space data using ESPIRiT [39], and outputs a refined estimate of the coil sensitivities (S). The IRN and CEN are trained jointly by multiplying the subspace images and sensitivity maps. Then the resulting multichannel images are multiplied by $V_{k}^{*}$ to yield time‐series images, and spiral sampling is performed using the NUFFT. Both networks are updated by computing the mean square error (MSE) loss between the reconstructed and acquired measurements data (denoted by y) at the sampled k‐space locations, after multiplication by the spiral density compensation function w (Equation 4).

(4) $${\underset{\theta_{\mathrm{IRN}},\theta_{\mathrm{CEN}}}{\arg\;\min}\left\|w\left(y-\mathrm{FS}\left(xV_{k}^{*}\right)\right)\right\|}_{2}^{2}$$

In parallel, a third untrained network termed the parameter estimation network (PEN) estimates T_1_, T_2_, and M_0_ maps from the subspace images (Figure S4). The PEN is implemented as a fully‐connected network ($\theta_{\mathrm{PEN}}$) with two hidden layers having 300 nodes each. It operates on each voxel independently, taking as input a vector of length 2k (corresponding to the real and imaginary parts of the subspace images) and estimating T_1_, T_2_, and the real and imaginary components of the M_0_ scaling factor. To train the PEN (Equation 5), fingerprints are simulated for each voxel in the estimated T_1_ and T_2_ maps, scaled by the M_0_ maps, and projected onto the subspace (by multiplication with $V_{k}$) to generate synthetic images, which are compared to the images generated by the IRN using an MSE loss, as shown in Equation (5). During this step, the fingerprint simulation is done using a pre‐trained (frozen) network called the Fingerprint Generator Network (FGN). The FGN is a fully‐connected network that outputs simulated fingerprints given T_1_ values, T_2_ values, and cardiac RR interval timings as inputs. It serves as a fast approximation of the Bloch equations, avoiding the need to perform a time‐consuming Bloch equation simulation at every iteration of the DIP training [40].

(5) $$\arg{\min_{\theta_{\mathrm{PEN}}}\left\|x-M_{0}\left(\theta_{\mathrm{FGN}}\left(T_{1},T_{2},\mathrm{RR}\right)V_{k}\right)\right\|}_{2}^{2}$$

DIP‐MRF was implemented in TensorFlow with a Keras backend and run on a high‐performance cluster, reserving a single NVIDIA A40 GPU. Training was performed with an Adam optimizer, a learning rate of 0.001, a mini‐batch size of 16 TRs, and 300 epochs, with one epoch defined as a full pass over all TRs.

### 2.3 Phantom Experiments

Low‐field cardiac MRF was initially validated using the ISMRM/NIST MRI System Phantom which includes 14 vials with T_1_ values ranging from 67 to 1986 ms and T_2_ values ranging from 14 to 1044 ms at 0.55T [41]. Data were acquired on a 0.55T scanner using 6 channels from a brain phased array coil with a single slice positioned through the T_2_ layer of the phantom. An ECG signal simulating a constant heart rate of 60 beats per minute was used for prospective triggering. MRF data were collected using the proposed sequence (FOV 300 × 300 mm^2^, 192 × 192 matrix, spatial resolution 1.6 × 1.6 × 8.0 mm^3^) and reconstructed with both SLLR‐MRF and DIP‐MRF methods. For comparison, conventional cardiac mapping sequences were also acquired using MOLLI (5) and T_2_‐bSSFP (7) using sequence parameters optimized for 0.55T as described by Varghese et al. [42]. MOLLI data were acquired with a 4(1)3(1)2(1)2 sampling scheme within a 14‐heartbeat breathhold. T_2_‐bSSFP data were acquired with T_2_‐preparation times of 0, 25, and 60 ms, repeated twice (yielding six images) with two recovery heartbeats between each image within a 16‐heartbeat breathhold. Both conventional mapping sequences employed the same FOV (300 × 300 mm^2^) as MRF but a lower spatial resolution (2.1 × 2.1 × 8.0 mm^3^, matrix size 144 × 144), with zero‐filling interpolation applied to achieve a reconstructed in‐plane resolution of 1.0 × 1.0 mm^2^. The diastolic acquisition windows are 209 ms for MOLLI and 169 ms for T_2_‐bSSFP. Additional sequence parameters are provided in Table S1.

Reference T_1_ values were obtained using an inversion recovery sequence, while reference T_2_ values were measured with a single‐echo spin echo sequence. Regions of interests (ROIs) were manually delineated for each spherical compartment in the phantom, and the mean and standard deviation (SD) of T_1_ and T_2_ were computed within each ROI. In the T_2_ maps, spheres with reference values above 300 ms were excluded from analysis, as such values are not physiologically relevant for cardiac mapping and fall outside the intended measurement range of the MRF sequence.

### 2.4 Healthy Subject Imaging

Eighteen healthy subjects (11 female; ages 28 ± 8 years) were scanned at 0.55T after obtaining written informed consent in this HIPAA‐compliant, IRB‐approved study. Imaging was performed using a 12‐channel cardiac phased array coil and 6 channels from the spine array. MRF scans were acquired in all subjects from apical, mid, and basal short‐axis slices of the left ventricle (LV), with each slice imaged during an end‐expiratory breathhold using the same sequence parameters as in the phantom experiments. Maps were reconstructed using both SLLR‐MRF and DIP‐MRF. In seven subjects, scans were also acquired using conventional MOLLI and T_2_‐bSSFP sequences (Table S1). ECG triggering was performed with an external device (Expression MR 400, Philips N.V., Amsterdam, the Netherlands).

A known challenge with zero‐shot techniques such as DIP is the potential of overfitting to noise and aliasing artifacts. To mitigate this issue, dropout regularization was incorporated before each convolutional layer in the IRN, as in previous studies [8]. In one subject, reconstructions were performed using dropout rates ranging from 6% to 24% to empirically select the best value and evaluate its impact on map quality and quantitative T_1_ and T_2_ values. For all other subjects, a dropout rate of 18% was used based on these results.

### 2.5 Image Analysis

MRF and conventional maps were analyzed by manually delineating ROIs in myocardial segments based on the standardized American Heart Association (AHA) model [43]. Segmentations were performed by Zh. L. and reviewed by J.H., who have 3 and 13 years of experience in cardiac MRI, respectively. The mean T_1_ and T_2_ values were measured within each segment. In addition, T_1_ and T_2_ values were measured in the left (LV) and right ventricular (RV) blood pool from a mid‐ventricular slice.

Qualitative evaluation of the reconstructed maps was performed by three cardiac imaging clinicians with 2 (C.S.), 3 (W.T.), and 4 (M.G.) years of cardiac MRI reading experience, respectively. All maps were anonymized and presented in random order to the reviewers. Each map was independently assessed based on five criteria: (1) overall diagnostic quality, (2) absence of artifacts, (3) sharpness of the endocardial border, (4) sharpness of the epicardial border, and (5) apparent SNR. Each criterion was scored using a 5‐point Likert scale: 1 = non‐diagnostic, 5 = excellent. Scoring was performed for each of the three mapping methods (conventional scans, SLLR‐MRF, and DIP‐MRF) and was conducted separately for the T_1_ and T_2_ maps.

### 2.6 Statistical Analysis

In phantom studies, the precision of T_1_ and T_2_ values was quantified by measuring the coefficient of variation (COV). For both MRF and conventional sequences, the agreement in mean T_1_ and T_2_ values relative to reference values was assessed using linear regression.

Intrasubject variability was assessed by computing the mean of the means and standard deviations (SD) within each segment across all subjects. Intersubject variability was evaluated by calculating the SDs of the segment‐wise mean values across all subjects. Differences in T_1_ and T_2_ values between conventional scans, SLLR‐MRF, and DIP‐MRF were evaluated using a one‐way ANOVA followed by pairwise *t*‐tests with Bonferroni correction for multiple comparisons.

Differences in image ratings were assessed using a Friedman test followed by pairwise Wilcoxon signed‐rank tests with Bonferroni correction. Inter‐reader agreement was quantified using the intraclass correlation coefficient (ICC). A *p* value < 0.05 was considered significant.

## 3 Results

### 3.1 Phantom Experiments

**FIGURE 1:**
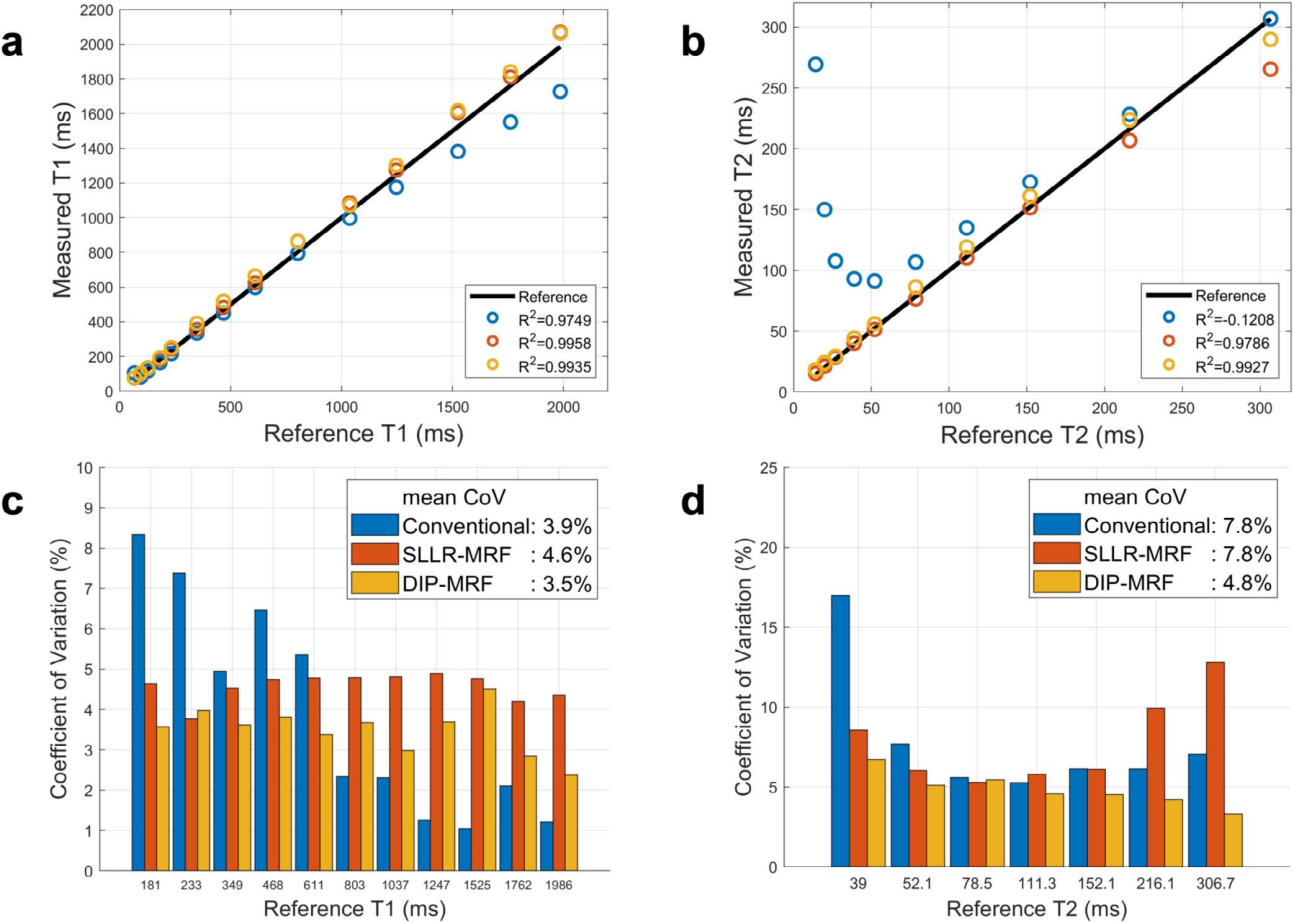
NIST phantom results using conventional cardiac mapping and MRF (with both SLLR‐MRF and DIP‐MRF reconstructions) at 0.55T. Linear regression plots are presented for phantom (a) T_1_ and (b) T_2_ measurements using conventional mapping (MOLLI or T_2_‐prepared bSSFP), SLLR‐MRF, and DIP‐MRF compared to reference standard sequences. (c) The coefficient of variation (COV), a measure of precision, is displayed for T_1_ and (d) T_2_ measurements within each phantom compartment. The values in the legend report the average COV across all compartments in the phantom.

Linear regression analyses showed strong agreement between the measured and reference T_1_ values across all methods (Figure 1). Conventional T_1_ mapping exhibited a tendency to underestimate higher T_1_ values, particularly those above 1000 ms. In contrast, MRF with both reconstruction approaches demonstrated excellent accuracy, with coefficients of determination (*R* ^2^) above 0.99. Conventional T_2_‐bSSFP mapping substantially overestimated short T_2_ values (below 100 ms). MRF exhibited better accuracy compared with reference T_2_ values, with DIP‐MRF outperforming the SLLR‐MRF reconstruction (*R* ^2^ = 0.993 vs. *R* ^2^ = 0.979). In terms of precision, DIP‐MRF exhibited a consistently lower COV across all compartments in the phantom, with a mean COV of 3.5% for T_1_ (excluding the first three vials with short T_1_ below 180 ms) and 4.8% for T_2_ (excluding vials with T_2_ below 30 ms or above 300 ms). Conventional methods had slightly higher mean COV values (3.9% for T_1_, 7.8% for T_2_), whereas SLLR‐MRF showed the highest variability with mean COV values of 4.6% for T_1_ and 7.8% for T_2_.

### 3.2 In Vivo Experiments

**FIGURE 2:**
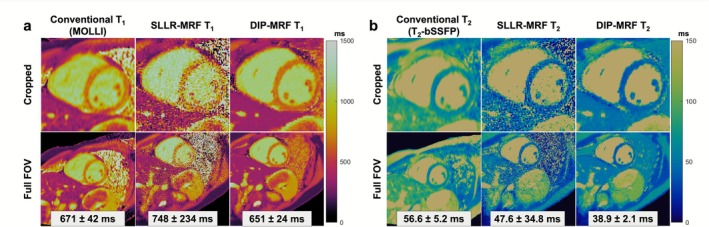
Representative 0.55T cardiac T_1_ and T_2_ maps using MRF and conventional mapping techniques. (a) T_1_ and (b) T_2_ maps are shown from conventional (MOLLI or T_2_‐prepared bSSFP) and MRF acquisitions. The same MRF data were reconstructed using both SLLR‐MRF and DIP‐MRF techniques. Measured relaxation times (reported as mean ± SD) over the myocardium are displayed as insets. Maps are shown (bottom row) over the full FOV and (top row) cropped to a zoomed‐in region over the heart.

**FIGURE 3:**
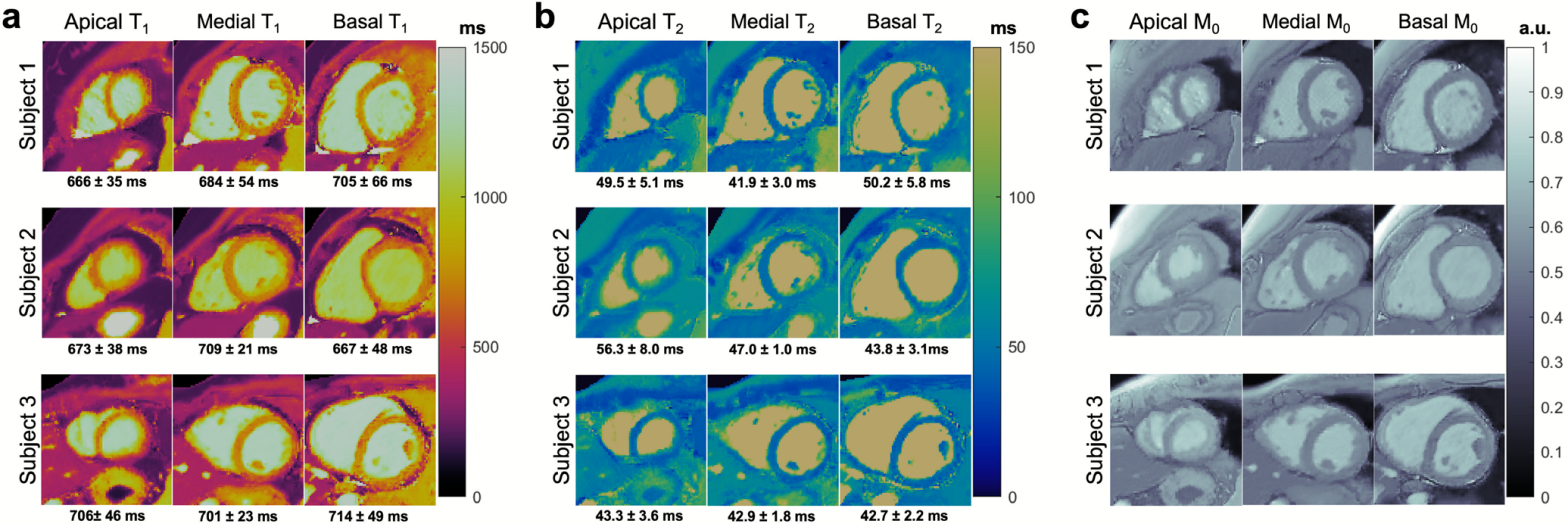
0.55T cardiac DIP‐MRF maps from three additional healthy subjects. (a) T_1_, (b) T_2_, and (c) M_0_ maps are shown from apical, mid, and basal short‐axis slice locations. Myocardial relaxation times (reported as mean ± SD) are provided in the figure.

Representative T_1_ and T_2_ maps from a mid‐ventricular short‐axis slice of a healthy subject are shown in Figure 2 using conventional mapping, SLLR‐MRF, and DIP‐MRF. Conventional T_1_ and T_2_ maps demonstrated greater spatial blurring compared to both SLLR‐MRF and DIP‐MRF reconstructions. DIP‐MRF yielded substantially lower noise than SLLR‐MRF, as reflected by the reduced standard deviations within the LV septum (234 vs. 24 ms for T_1_, and 34.8 vs. 2.1 ms for T_2_). Moreover, the standard deviations with DIP‐MRF were also lower than those with conventional mapping (42 ms for T_1_ and 5.2 ms for T_2_). DIP‐MRF maps from three additional healthy subjects are shown in Figure 3 across apical, mid, and basal short‐axis slices. Additionally, for comparison, maps reconstructed using NUFFT gridding followed by dictionary matching are provided in Figure S5, which exhibited severe noise enhancement.

**FIGURE 4:**
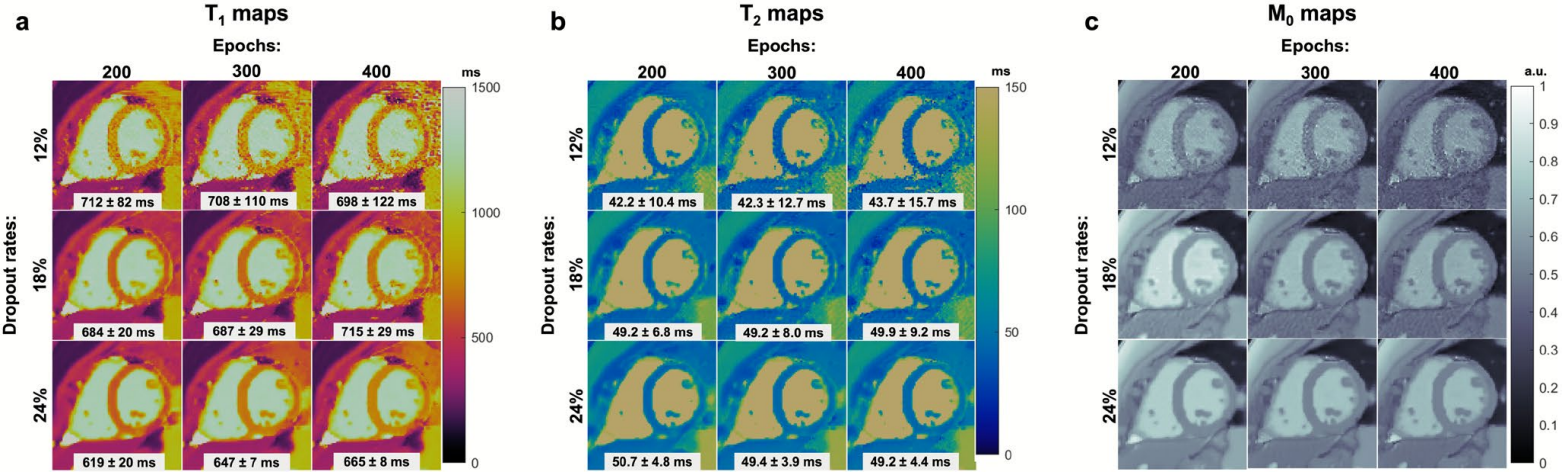
Impact of dropout rate and training epochs on DIP‐MRF reconstruction stability. (a) T_1_ maps, (b) T_2_ maps, and (c) M_0_ maps reconstructed at 200, 300, and 400 epochs with dropout rates of 12%, 18%, and 24%. Higher dropout rates reduce variability but can lead to underestimation of T_1_ values, whereas moderate dropout (18%) provides stable estimates with low standard deviation. Lower dropout rates amplify noise. At lower training epochs, residual artifacts remain, while at higher epochs the network begins to overfit noise. M_0_ maps remain robust across training epochs, indicating consistent reconstruction performance.

**FIGURE 5:**
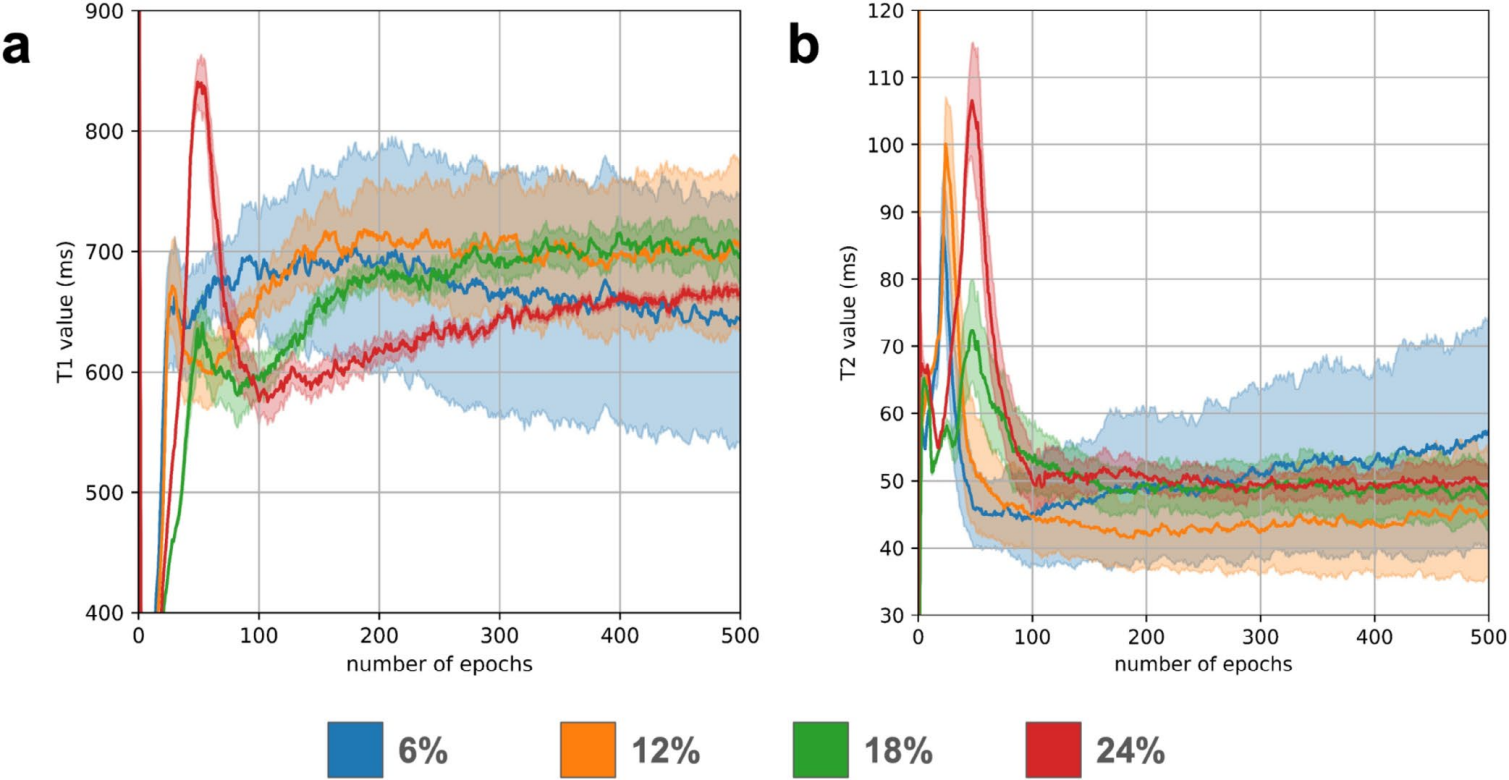
Effect of dropout rate and number of training epochs on myocardial relaxation times using DIP‐MRF. (a) T_1_ and (b) T_2_ values measured in the LV septum in one subject are plotted as a function of training epochs for DIP‐MRF using dropout rates of 6%, 12%, 18%, and 24%. Shaded regions indicate the T_1_ and T_2_ standard deviation within the LV septum. Based on these results, all subsequent reconstructions employed 18% dropout with 300 training epochs.

The effects of varying the dropout rate and training epochs in the DIP‐MRF reconstruction are shown in Figure 4 (Full FOV maps shown in Figure S6). Noise enhancement due to overfitting was observed when training for too many epochs (e.g., 400 epochs) or when the dropout rate was set too low (e.g., 12%). In contrast, the maps exhibited spatial blurring, indicative of underfitting, when too few training epochs were used (e.g., 200 epochs) or when the dropout rate was too high (e.g., 24%). A moderate dropout rate of 18% with 300 training epochs was empirically determined to yield the best map quality, enabling delineation of fine anatomical structures like the papillary muscles while limiting noise enhancement. This selection is justified quantitatively in Figure 5, which plots the mean and SD of myocardial T_1_ and T_2_ for this subject as a function of training epochs for different dropout rates. The mean myocardial T_1_ and T_2_ stabilize after approximately 300 epochs with 18% dropout. Lower dropout rates led to higher standard deviations, while higher dropout rates required substantially more epochs for the mean values to converge.

**FIGURE 6:**
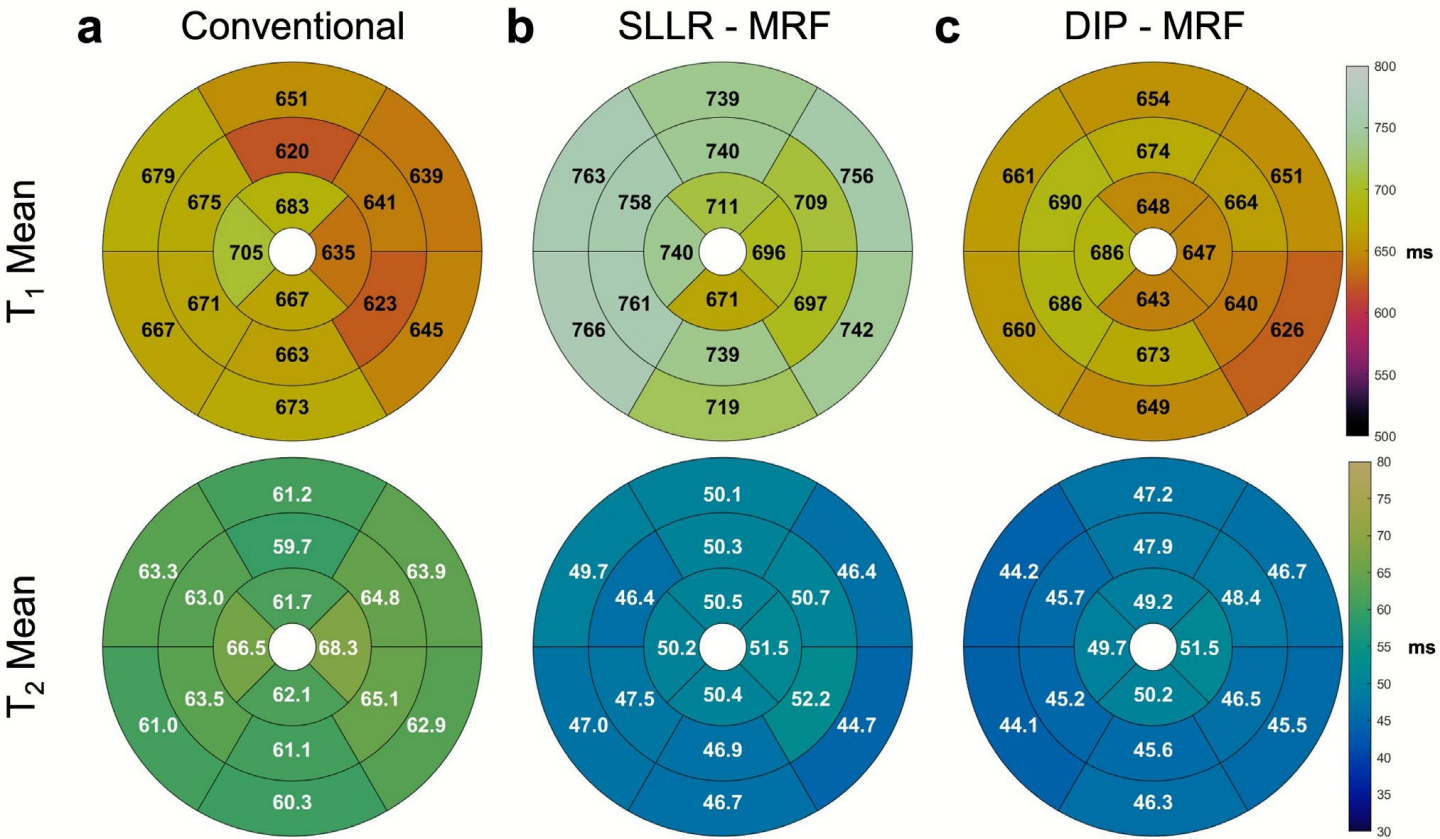
AHA 16‐segment bullseye plots showing mean T_1_ and T_2_ values across healthy subjects. Results are presented for (a) conventional MOLLI and T_2_‐prepared sequences, (b) SLLR‐MRF, and (c) DIP‐MRF. Note that MRF data were acquired in all 18 subjects, whereas conventional mapping was only obtained in 7 subjects.

**FIGURE 7:**
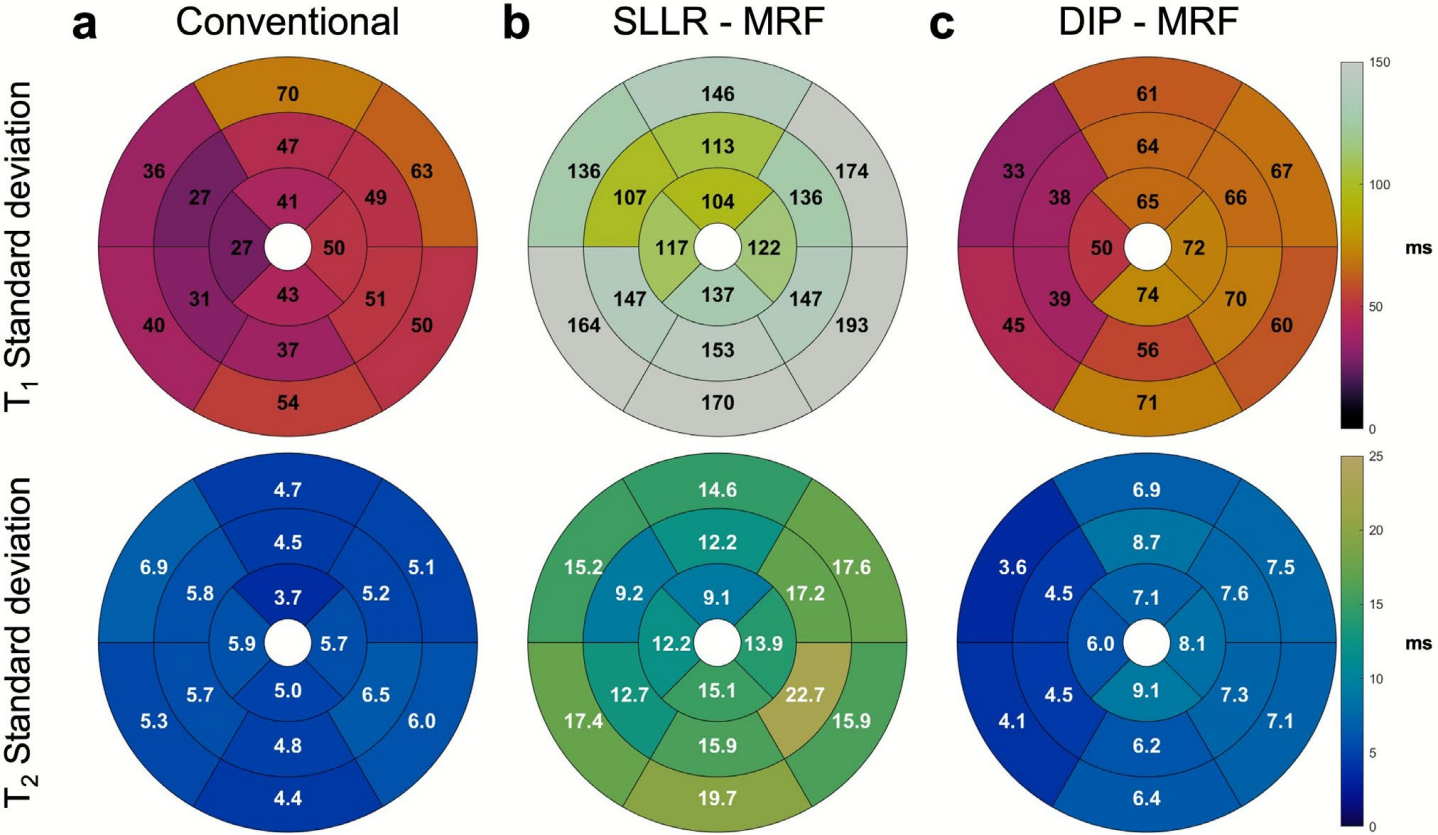
AHA 16‐segment bullseye plots showing T_1_ and T_2_ standard deviations. Results are presented for (a) conventional MOLLI and T_2_‐prepared sequences, (b) SLLR‐MRF, and (c) DIP‐MRF. The values represent the intrasubject variability, calculated by first measuring the within‐segment standard deviation in each subject, and then over all subjects. Note that MRF data were acquired in all 18 subjects, whereas conventional mapping was only obtained in 7 subjects.

AHA 16‐segment bullseye plots summarizing the mean and intrasubject variability of mean and SD T_1_ and T_2_ values across all subjects for each method are shown in Figures 6 and 7, respectively. For T_1_, SLLR‐MRF produced consistently higher mean values, while DIP‐MRF and MOLLI yielded comparable values. For example, in the LV septum (segment #9), mean T_1_ values were 671 ms with MOLLI, 761 ms with SLLR‐MRF, and 686 ms with DIP‐MRF. For T_2_, T_2_‐bSSFP yielded the highest mean values across all segments, followed by SLLR‐MRF, with DIP‐MRF producing slightly lower values than SLLR‐MRF. For example, in the LV septum (segment #9), mean T_2_ values were 63.5 ms with T_2_‐bSSFP, 47.5 with SLLR‐MRF, and 45.2 ms with DIP‐MRF. T_1_ and T_2_ measurements in the LV and RV blood pool are reported in Tables S2 and S3. Across all subjects, DIP‐MRF yielded blood T_1_ values of 1213 ± 30 ms (LV) and 1213 ± 56 ms (RV), and blood T_2_ values of 196.6 ± 9.3 ms (LV) and 198.0 ± 19.5 ms (RV). Significant differences were observed in RV blood T_1_ between the MOLLI and MRF‐based methods. For T_2_, both SLLR‐MRF and DIP‐MRF showed significant difference in the LV and RV blood pools.

In terms of variability, the intrasubject SDs of myocardial T_1_ values from DIP‐MRF and MOLLI were similar (between 30 and 70 ms for all segments). With both methods, lower variability was observed in the septal regions than in the lateral wall. SLLR‐MRF showed substantially greater variability, with T_1_ SDs exceeding 100 ms in all segments. For T_2_ measurements, both DIP‐MRF and T_2_‐bSSFP achieved low variability, with SDs ranging from 4 to 9 ms in all segments. SLLR‐MRF again demonstrated the highest variability, with T_2_ SDs typically exceeding 10 ms across segments.

**FIGURE 8:**
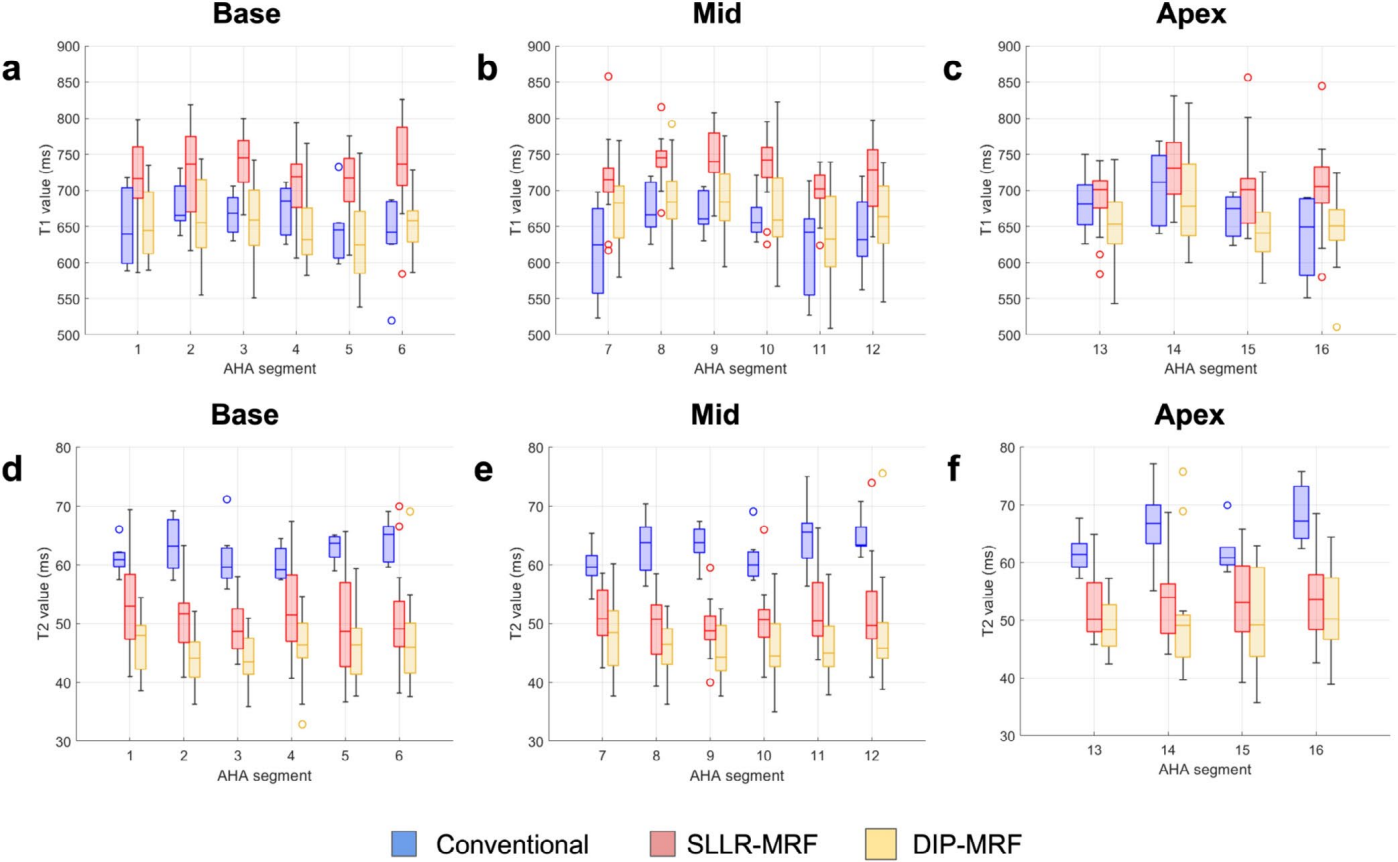
Segmental comparison of myocardial T_1_ and T_2_ values obtained from three reconstruction methods across the 16 standardized AHA segments. (a–c) T_1_ values (ms) for basal (segments 1–6), mid‐ventricular (segments 7–12), and apical (segments 13–16) regions. (d–f) T_2_ values (ms) for the same corresponding regions. Results are shown for conventional mapping (blue), SLLR‐MRF (red), and DIP‐MRF (yellow). Boxplots represent the median (central line), interquartile range (boxes), and full data spread excluding outliers (whiskers), with outliers displayed as individual points. Statistical comparison indicates that SLLR‐MRF T_1_ values differ significantly from both conventional mapping and DIP‐MRF, while DIP‐MRF shows significant differences from conventional mapping in selected segments. For T_2_, all three methods demonstrate significant differences across all myocardial segments.

Figure 8 presents boxplots summarizing the mean and intersubject variability of T_1_ and T_2_ values within each myocardial segment for conventional mapping, SLLR‐MRF, and DIP‐MRF. The trends in mean values mirrored those observed in the bullseye plots. SLLR‐MRF yielded significantly higher T_1_ values than MOLLI in most segments (excluding segments 11–16) and higher than DIP‐MRF in all segments, while MOLLI and DIP‐MRF yielded comparable values (with significant differences only in segment 15). DIP‐MRF yielded significantly lower T_2_ values compared to T_2_‐bSSFP in all segments and compared to SLLR‐MRF in most segments (Table S4). SLLR‐MRF showed greater intersubject variability than conventional mapping, while DIP‐MRF had similar variability to conventional mapping.

**FIGURE 9:**
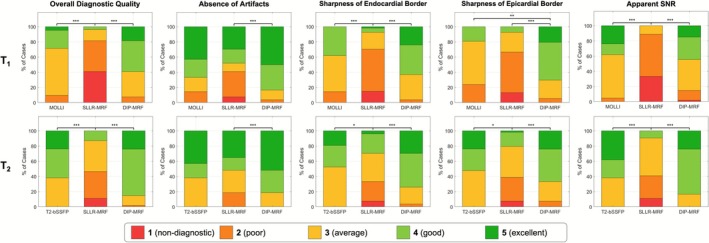
Qualitative evaluation of T_1_ (top row) and T_2_ (bottom row) maps by three independent radiologists. Image quality was scored on a 5‐point Likert scale (1 = non‐diagnostic, 2 = poor, 3 = average, 4 = good, 5 = excellent) across five categories: Overall diagnostic quality, absence of artifacts, sharpness of the endocardial border, sharpness of the epicardial border, and apparent SNR. T_1_ comparisons were performed among MOLLI, SLLR‐MRF, and DIP‐MRF, while T_2_ comparisons were made among T_2_‐bSSFP, SLLR‐MRF, and DIP‐MRF. The y‐axis represents the percentage of all cases (i.e., the number of cases assigned a given score divided by the total number of cases). DIP‐MRF consistently achieved higher diagnostic ratings and significantly outperformed SLLR‐MRF across all categories, with image quality comparable to or better than conventional reference methods. Asterisks indicate statistical significance (*p < 0.05, **p < 0.01, ***p < 0.001).

The expert image quality rating results are shown in Figure 9. The height of each color‐coded column represents the relative proportion of times the maps were rated with a particular score. For overall image quality, the mean ratings were 3.4 (MOLLI), 2.3 (SLLR‐MRF), and 3.8 (DIP‐MRF) for T_1_, and 3.9 (T_2_‐bSSFP), 2.9 (SLLR‐MRF), and 4.1 (DIP‐MRF) for T_2_. Both MOLLI and DIP‐MRF T_1_ maps received significantly higher ratings than SLLR‐MRF in overall image quality, absence of artifacts, sharpness of endocardial borders, and apparent SNR. There were no significant differences in scores between DIP‐MRF and MOLLI in most categories (Table S5), with the exception of sharpness of epicardial borders, where DIP‐MRF was rated significantly higher (3.0 vs. 3.9). For T_2_ maps, there were no significant differences in scores between DIP‐MRF and T_2_‐bSSFP, and both methods were rated significantly higher than SLLR‐MRF in all categories. While not statistically significant, DIP‐MRF exhibited higher inter‐reader agreement than conventional sequences and was higher for T_1_ mapping compared to T_2_ mapping. Across all methods, ICC values for overall image quality were 0.74 (MOLLI), 0.77 (SLLR‐MRF), and 0.88 (DIP‐MRF) for T_1_ maps and 0.52 (T_2_‐bSSFP), 0.81 (SLLR‐MRF), and 0.71 (DIP‐MRF) for T_2_ maps.

## 4 Discussion

This study demonstrated the feasibility of performing simultaneous 2D cardiac T_1_, T_2_, and M_0_ mapping during a single breathhold using cardiac MRF on a commercial 0.55T MRI scanner. The MRF sequence was adapted from previous implementations developed at higher field strengths, with modifications to the spiral readout to accommodate the reduced gradient performance of the low‐field system. As a result, fewer total time points were collected compared to previous higher‐field studies to maintain a clinically feasible breathhold duration and diastolic acquisition window. The combination of fewer time points and the inherently lower SNR at 0.55T necessitated the use of reconstruction methods beyond simple gridding and dictionary matching, the use of which led to severe noise enhancement.

Two reconstruction methods with denoising capabilities were investigated: (1) SLLR‐MRF and (2) DIP‐MRF. In phantom experiments, both methods produced T_1_ and T_2_ values in good agreement with reference measurements, with DIP‐MRF yielding better precision, as indicated by overall lower COV values. In vivo, DIP‐MRF provided better delineation of fine anatomical structures and more effective noise suppression, resulting in more homogeneous T_1_ and T_2_ maps with smaller SD values across all myocardial segments.

In previous studies at 1.5 and 3 T, MRF T_1_ values were typically higher than those from MOLLI, which is known to underestimate myocardial T_1_ (9). While DIP‐MRF yielded slightly higher T_1_ values than MOLLI in this study, the magnitude of this difference was small and not significant, with a mean T_1_ of 686 ms using DIP‐MRF and 671 ms using MOLLI in the LV septum, close to literature values of 701 ms for MOLLI at 0.55T [23]. While MOLLI is well‐known to underestimate T_1_ values at 1.5 and 3T, these effects may be partially mitigated at 0.55T, as MOLLI accuracy improves in regimes with shorter T_1_ and longer T_2_, the shorter T_1_ values allow for more complete recovery of longitudinal magnetization between inversions. This interpretation is supported by recent work at 0.55 T by Sotta et al. [44], who reported that T_1_ mapping estimates using a dictionary‐based technique called saturation‐recovery and variable‐flip‐angle (SAVA) were comparable to those from MOLLI.

Similar to findings at higher field strengths, DIP‐MRF yielded shorter T_2_ values than conventional T_2_‐bSSFP mapping (45.2 vs. 63.5 ms), as well as literature values (58 ms) for T_2_‐bSSFP at 0.55T [23]. This is consistent with the known overestimation of T_2_ values by T_2_‐bSSFP, an effect confirmed in phantom experiments where MRF more closely matched reference spin echo values, whereas T_2_‐bSSFP systematically overestimated T_2_ values, particularly for regions with T_2_ < 100 ms (a range that includes expected values in myocardium). In addition, prior studies using FISP‐based MRF sequences in various organs, including the heart, have reported lower in vivo T_2_ values relative to reference methods, potentially due to motion sensitivity along the direction of the unbalanced gradient (the slice‐selection axis), magnetization transfer effects [45], or intravoxel dephasing [46].

This study employed a FISP‐based MRF sequence that has been widely adopted for body imaging applications due to its low sensitivity to off‐resonance effects [16, 35], thereby minimizing banding artifacts and eliminating the need to model off‐resonance as an additional property in the dictionary. An alternative approach would be to employ a bSSFP readout, which offers higher SNR efficiency and could benefit from the improved B_0_ homogeneity at low field. Nevertheless, while bSSFP banding artifacts may be reduced at 0.55T, notable off‐resonance frequency shifts can still occur across the heart. Kellman et al. reported > 80 Hz off‐resonance across the LV of some subjects at 1.5T even after cardiac shimming, which would scale to > 27 Hz at 0.55T [47]. Because the bSSFP MRF signal evolution is sensitive to even small frequency offsets [15], accurate T_1_ and T_2_ quantification may still require modeling off‐resonance as an additional dimension of the dictionary, even at 0.55T, increasing its size and computational complexity.

Recently, promising results using cardiac MRF for T_1_, T_2_, and fat fraction mapping have been demonstrated at 0.55T [48]. While fat fraction was not included in the present study, there are several key differences worth noting: (1) that study employed a bSSFP readout (without B_0_ estimation) compared to the FISP readout in this study, with advantages and limitations as discussed above; (2) the current study achieved a higher spatial resolution (1.6 × 1.6 × 8.0 mm^3^ vs. 2.0 × 2.0 × 10.0 mm^3^); (3) a physics‐guided deep learning (DIP‐MRF) reconstruction was used here to address the challenge of low SNR at 0.55T, as opposed to a low‐rank tensor (HD‐PROST) reconstruction; (4) the current study includes image quality ratings comparing MRF and conventional techniques.

## 5 Limitations

First, DIP‐MRF is computationally intensive, as training is performed from scratch for each dataset, and requires approximately 30 min per slice on an NVIDIA A40 GPU, which is currently a barrier to clinical deployment. This computational bottleneck could potentially be addressed by initializing the U‐Net weights using MRF data from multiple subjects instead of training de novo. For each new reconstruction, the initialized weights could then be rapidly fine‐tuned for an individual dataset using the forward physics model to enforce data consistency [49]. Second, the breathhold duration and diastolic acquisition window may be long for some patients, increasing susceptibility to respiratory or cardiac motion artifacts. This could potentially be addressed by numerically optimizing the flip angle pattern and preparation pulses specifically for 0.55T relaxation properties, which could enable a more efficient MRF acquisition [50, 51, 52, 53]. Third, unlike the conventional mapping sequences, MRF currently does not incorporate motion correction. Fourth, the spiral trajectory employed in this study could be further optimized to improve scan efficiency and SNR at 0.55T by leveraging the lengthened T_2_* values at 0.55T [23]. There is likely a tradeoff between spiral readout length and quantitative accuracy, as longer readouts would reduce aliasing artifacts in MRF time‐series images but permit collection of fewer total images within a fixed breathhold duration and cardiac acquisition window, which could compromise quantitative accuracy. Finally, this study was limited to a relatively small sample size, with 18 subjects undergoing MRF and a smaller subset of only 7 subjects undergoing comparisons with conventional mapping. Furthermore, no cardiac patients were included in this study. Future validation in post‐contrast settings and in patient populations are essential next steps.

## 6 Conclusion

This study demonstrated the feasibility of performing simultaneous cardiac T_1_, T_2_, and M_0_ mapping using MRF with DIP reconstruction in healthy subjects on a commercial 0.55T scanner. The denoising capabilities of DIP‐MRF resulted in improved measurement precision and superior ratings of overall image quality compared to MRF with a low‐rank (SLLR‐MRF) reconstruction, as well as conventional MOLLI and T_2_‐bSSFP mapping.

## Funding

This work was supported by NIH (NHLBI R01HL163030), NSF (IIS‐2435746), DARPA (grant number HR00112520042), MICDE Catalyst Grant, MIDAS PODS Grant, and Siemens Healthineers.

## Supporting information


**Data S1:** jmri70239‐sup‐0001‐Supinfo.docx.

## References

[1] D. R. Messroghli , J. C. Moon , V. M. Ferreira , et al., “Clinical Recommendations for Cardiovascular Magnetic Resonance Mapping of T1, T2, T2* and Extracellular Volume: A Consensus Statement by the Society for Cardiovascular Magnetic Resonance (SCMR) Endorsed by the European Association for Cardiovascular Imaging (EACVI),” Journal of Cardiovascular Magnetic Resonance 19 (2016): 75. 10.1186/s12968-017-0389-8 PMC5633041 28992817

[2] A. J. Taylor , M. Salerno , R. Dharmakumar , and M. Jerosch‐Herold , “T1 Mapping: Basic Techniques and Clinical Applications,” JACC: Cardiovascular Imaging 9 (2016): 67–81. 26762877 10.1016/j.jcmg.2015.11.005

[3] V. O. Puntmann , E. Peker , Y. Chandrashekhar , and E. Nagel , “T1 Mapping in Characterizing Myocardial Disease,” Circulation Research 119 (2016): 277–299. 27390332 10.1161/CIRCRESAHA.116.307974

[4] A. T. O'Brien , K. E. Gil , J. Varghese , O. P. Simonetti , and K. M. Zareba , “T2 Mapping in Myocardial Disease: A Comprehensive Review,” Journal of Cardiovascular Magnetic Resonance 24 (2022): 33. 35659266 10.1186/s12968-022-00866-0 PMC9167641

[5] D. R. Messroghli , A. Radjenovic , S. Kozerke , D. M. Higgins , M. U. Sivananthan , and J. P. Ridgway , “Modified Look‐Locker Inversion Recovery (MOLLI) for High‐Resolution T1 Mapping of the Heart,” Magnetic Resonance in Medicine 52 (2004): 141–146. 15236377 10.1002/mrm.20110

[6] K. Chow , J. A. Flewitt , J. D. Green , J. J. Pagano , M. G. Friedrich , and R. B. Thompson , “Saturation Recovery Single‐Shot Acquisition (SASHA) for Myocardial T1 Mapping,” Magnetic Resonance in Medicine 71 (2014): 2082–2095. 23881866 10.1002/mrm.24878

[7] S. Giri , Y.‐C. Chung , A. Merchant , et al., “T2 Quantification for Improved Detection of Myocardial Edema,” Journal of Cardiovascular Magnetic Resonance 11 (2009): 56. 20042111 10.1186/1532-429X-11-56 PMC2809052

[8] J. I. Hamilton , Y. Jiang , D. Ma , et al., “Investigating and Reducing the Effects of Confounding Factors for Robust T1 and T2 Mapping With Cardiac MR Fingerprinting,” Magnetic Resonance Imaging 53 (2018): 40–51. 29964183 10.1016/j.mri.2018.06.018 PMC7755105

[9] P. Kellman and M. S. Hansen , “T1‐Mapping in the Heart: Accuracy and Precision,” Journal of Cardiovascular Magnetic Resonance 16 (2014): 2. 24387626 10.1186/1532-429X-16-2 PMC3927683

[10] S. Kvernby , M. J. B. Warntjes , H. Haraldsson , C.‐J. Carlhäll , J. Engvall , and T. Ebbers , “Simultaneous Three‐Dimensional Myocardial T1 and T2 Mapping in One Breath Hold With 3D‐QALAS,” Journal of Cardiovascular Magnetic Resonance 16 (2014): 102. 25526880 10.1186/s12968-014-0102-0 PMC4272556

[11] A. G. Christodoulou , J. L. Shaw , C. Nguyen , et al., “Magnetic Resonance Multitasking for Motion‐Resolved Quantitative Cardiovascular Imaging,” Nature Biomedical Engineering 2 (2018): 215–226. 10.1038/s41551-018-0217-y PMC6141200 30237910

[12] K. Chow , G. Hayes , J. A. Flewitt , et al., “Improved Accuracy and Precision With Three‐Parameter Simultaneous Myocardial T1 and T2 Mapping Using Multiparametric SASHA,” Magnetic Resonance in Medicine 87 (2022): 2775–2791. 35133018 10.1002/mrm.29170

[13] M. Henningsson , “Cartesian Dictionary‐Based Native T1 and T2 Mapping of the Myocardium,” Magnetic Resonance in Medicine 87 (2022): 2347–2362. 34985143 10.1002/mrm.29143

[14] Z. Lyu , S. Hua , J. Xu , et al., “Free‐Breathing Simultaneous Native Myocardial T1, T2 and T1ρ Mapping With Cartesian Acquisition and Dictionary Matching,” Journal of Cardiovascular Magnetic Resonance 25 (2023): 63. 37946191 10.1186/s12968-023-00973-6 PMC10636995

[15] D. Ma , V. Gulani , N. Seiberlich , et al., “Magnetic Resonance Fingerprinting,” Nature 495 (2013): 187–192. 23486058 10.1038/nature11971 PMC3602925

[16] J. I. Hamilton , Y. Jiang , Y. Chen , et al., “MR Fingerprinting for Rapid Quantification of Myocardial T1, T2, and Proton Spin Density,” Magnetic Resonance in Medicine 77 (2017): 1446–1458. 27038043 10.1002/mrm.26216 PMC5045735

[17] J. I. Hamilton , Y. Jiang , B. Eck , M. Griswold , and N. Seiberlich , “Cardiac Cine Magnetic Resonance Fingerprinting for Combined Ejection Fraction, T1 and T2 Quantification,” NMR in Biomedicine 33 (2020): e4323. 32500541 10.1002/nbm.4323 PMC7772953

[18] Y. Liu , J. Hamilton , Y. Jiang , and N. Seiberlich , “Cardiac MRF Using Rosette Trajectories for Simultaneous Myocardial T1, T2, and Proton Density Fat Fraction Mapping,” Frontiers in Cardiovascular Medicine 9 (2022): 9. 10.3389/fcvm.2022.977603 PMC9530568 36204572

[19] M. Crabb , K. Kunze , D. Tripp , et al., “3D Whole‐Heart Joint T1/T1ρ/T2 Mapping and Water‐Fat Imaging for Contrast‐Agent Free Myocardial Tissue Characterization at 0.55T,” Journal of Cardiovascular Magnetic Resonance 26 (2024): 100113, 10.1016/j.jocmr.2024.100113.

[20] S. Kaplan , G. L. da Cruz , C. Madamanchi , et al., “Simultaneous T1, T2, and T1ρ Mapping of the Myocardium Using Cardiac MR Fingerprinting With a Deep Image Prior Reconstruction,” Magnetic Resonance in Medicine 94 (2025): 1500–1513. 40407793 10.1002/mrm.30580 PMC12309879

[21] E. Cummings , G. L. D. Cruz , J. Hamilton , and N. Seiberlich , “Cardiac T1, T2, T2*, and PDFF Mapping at 0.55T With Rosette MR Fingerprinting and a Deep Image Prior Reconstruction,” Journal of Cardiovascular Magnetic Resonance 27 (2025).

[22] T. C. Arnold , C. W. Freeman , B. Litt , and J. M. Stein , “Low‐Field MRI: Clinical Promise and Challenges,” Journal of Magnetic Resonance Imaging 57 (2023): 25–44. 36120962 10.1002/jmri.28408 PMC9771987

[23] A. E. Campbell‐Washburn , R. Ramasawmy , M. C. Restivo , et al., “Opportunities in Interventional and Diagnostic Imaging by Using High‐Performance Low‐Field‐Strength MRI,” Radiology 293 (2019): 384–393. 31573398 10.1148/radiol.2019190452 PMC6823617

[24] ISMRM , “A Comparison of Metal Artifacts in Cardiovascular MRI at 0.55T and 1.5T,” (2021), https://archive.ismrm.org/2021/3636.html.

[25] J. Varghese , J. Craft , C. D. Crabtree , et al., “Assessment of Cardiac Function, Blood Flow and Myocardial Tissue Relaxation Parameters at 0.35 T,” NMR in Biomedicine 33 (2020): e4317. 32363644 10.1002/nbm.4317

[26] D. Si , M. G. Crabb , K. P. Kunze , S. J. Littlewood , C. Prieto , and R. M. Botnar , “Free‐Breathing 3D Whole‐Heart Joint T1/T2 Mapping and Water/Fat Imaging at 0.55 T,” Magnetic Resonance in Medicine 92 (2024): 1511–1524. 38872384 10.1002/mrm.30139

[27] A. E. Campbell‐Washburn , J. Varghese , K. S. Nayak , R. Ramasawmy , and O. P. Simonetti , “Cardiac MRI at Low Field Strengths,” Journal of Magnetic Resonance Imaging 59 (2024): 412–430. 37530545 10.1002/jmri.28890 PMC10834858

[28] C. C. Cline , X. Chen , B. Mailhe , et al., “AIR‐MRF: Accelerated Iterative Reconstruction for Magnetic Resonance Fingerprinting,” Magnetic Resonance Imaging 41 (2017): 29–40. 28716682 10.1016/j.mri.2017.07.007

[29] E. Y. Pierre , D. Ma , Y. Chen , C. Badve , and M. A. Griswold , “Multiscale Reconstruction for MR Fingerprinting,” Magnetic Resonance in Medicine 75 (2016): 2481–2492. 26132462 10.1002/mrm.25776 PMC4696924

[30] D. F. McGivney , E. Pierre , D. Ma , et al., “SVD Compression for Magnetic Resonance Fingerprinting in the Time Domain,” IEEE Transactions on Medical Imaging 33 (2014): 2311–2322. 25029380 10.1109/TMI.2014.2337321 PMC4753055

[31] G. Lima da Cruz , A. Bustin , O. Jaubert , T. Schneider , R. M. Botnar , and C. Prieto , “Sparsity and Locally Low Rank Regularization for MR Fingerprinting,” Magnetic Resonance in Medicine 81 (2019): 3530–3543. 30720209 10.1002/mrm.27665 PMC6492150

[32] J. I. Hamilton , “A Self‐Supervised Deep Learning Reconstruction for Shortening the Breathhold and Acquisition Window in Cardiac Magnetic Resonance Fingerprinting,” Frontiers in Cardiovascular Medicine 9 (2022): 9. 10.3389/fcvm.2022.928546 PMC9260051 35811730

[33] D. Ulyanov , A. Vedaldi , and V. Lempitsky , “Deep Image Prior,” International Journal of Computer Vision 128 (2018): 9446–9454.

[34] Z. Shi , P. Mettes , S. Maji , and C. G. M. Snoek , “On Measuring and Controlling the Spectral Bias of the Deep Image Prior,” International Journal of Computer Vision 130 (2022): 885–908.

[35] Y. Jiang , D. Ma , N. Seiberlich , V. Gulani , and M. A. Griswold , “MR Fingerprinting Using Fast Imaging With Steady State Precession (FISP) With Spiral Readout,” Magnetic Resonance in Medicine 74 (2015): 1621–1631. 25491018 10.1002/mrm.25559 PMC4461545

[36] J. H. Brittain , B. S. Hu , G. A. Wright , C. H. Meyer , A. Macovski , and D. G. Nishimura , “Coronary Angiography With Magnetization‐Prepared T2 Contrast,” Magnetic Resonance in Medicine 33 (1995): 689–696. 7596274 10.1002/mrm.1910330515

[37] J. I. Hamilton , S. Pahwa , J. Adedigba , et al., “Simultaneous Mapping of T1 and T2 Using Cardiac Magnetic Resonance Fingerprinting in a Cohort of Healthy Subjects at 1.5T,” Journal of Magnetic Resonance Imaging 52 (2020): 1044–1052. 32222092 10.1002/jmri.27155 PMC7772954

[38] J. A. Fessler and B. P. Sutton , “Nonuniform Fast Fourier Transforms Using Min‐Max Interpolation,” IEEE Transactions on Signal Processing 51 (2003): 560–574.

[39] M. Uecker , P. Lai , M. J. Murphy , et al., “ESPIRiT—An Eigenvalue Approach to Autocalibrating Parallel MRI: Where SENSE Meets GRAPPA,” Magnetic Resonance in Medicine 71 (2014): 990–1001. 23649942 10.1002/mrm.24751 PMC4142121

[40] J. I. Hamilton and N. Seiberlich , “Machine Learning for Rapid Magnetic Resonance Fingerprinting Tissue Property Quantification,” Proceedings of the IEEE 108 (2020): 69–85. 33132408 10.1109/JPROC.2019.2936998 PMC7595247

[41] K. F. Stupic , M. Ainslie , M. A. Boss , et al., “A Standard System Phantom for Magnetic Resonance Imaging,” Magnetic Resonance in Medicine 86 (2021): 1194–1211. 33847012 10.1002/mrm.28779 PMC8252537

[42] J. Varghese , N. Jin , D. Giese , et al., “Building a Comprehensive Cardiovascular Magnetic Resonance Exam on a Commercial 0.55 T System: A Pictorial Essay on Potential Applications,” Frontiers in Cardiovascular Medicine 10 (2023): 10. 10.3389/fcvm.2023.1120982 PMC10014600 36937932

[43] M. D. Cerqueira , N. J. Weissman , V. Dilsizian , et al., “Standardized Myocardial Segmentation and Nomenclature for Tomographic Imaging of the Heart: A Statement for Healthcare Professionals From the Cardiac Imaging Committee of the Council on Clinical Cardiology of the American Heart Association,” Circulation 105 (2002): 539–542. 11815441 10.1161/hc0402.102975

[44] R. I. la De Sotta , M. G. Crabb , K. P. Kunze , R. M. Botnar , and C. Prieto , “Motion Corrected 3D Whole‐Heart SAVA T1 Mapping at 0.55 T,” Magnetic Resonance in Medicine 95 (2026): 234–248. 40851297 10.1002/mrm.70038

[45] T. Hilbert , D. Xia , K. T. Block , et al., “Magnetization Transfer in Magnetic Resonance Fingerprinting,” Magnetic Resonance in Medicine 84 (2020): 128–141. 31762101 10.1002/mrm.28096 PMC7083689

[46] J. Assländer , S. J. Glaser , and J. Hennig , “Pseudo Steady‐State Free Precession for MR‐Fingerprinting,” Magnetic Resonance in Medicine 77 (2017): 1151–1161. 27079826 10.1002/mrm.26202

[47] P. Kellman , D. A. Herzka , A. E. Arai , and M. S. Hansen , “Influence of Off‐Resonance in Myocardial T1‐Mapping Using SSFP Based MOLLI Method,” Journal of Cardiovascular Magnetic Resonance 15 (2013): 63. 23875774 10.1186/1532-429X-15-63 PMC3733653

[48] D. Pedraza , C. Castillo‐Passi , K. Kunze , R. M. Botnar , and C. Prieto , “Cardiac Magnetic Resonance Fingerprinting for Simultaneous T1, T2, and Fat‐Fraction Quantification at 0.55 T,” NMR in Biomedicine 38 (2025): e70143. 40944613 10.1002/nbm.70143

[49] L. Shen , J. Pauly , and L. Xing , “NeRP: Implicit Neural Representation Learning With Prior Embedding for Sparsely Sampled Image Reconstruction,” IEEE Transactions on Neural Networks and Learning Systems 35 (2024): 770–782. 10.1109/TNNLS.2022.3177134 PMC10889906 35657845

[50] S. Hu , S. Jordan , R. Boyacioglu , et al., “A Fast MR Fingerprinting Simulator for Direct Error Estimation and Sequence Optimization,” Magnetic Resonance Imaging 98 (2023): 105–114. 36681312 10.1016/j.mri.2023.01.011 PMC10002151

[51] B. Zhao , J. P. Haldar , C. Liao , et al., “Optimal Experiment Design for Magnetic Resonance Fingerprinting: Cramér‐Rao Bound Meets Spin Dynamics,” IEEE Transactions on Medical Imaging 38 (2019): 844–861. 30295618 10.1109/TMI.2018.2873704 PMC6447464

[52] C. Slioussarenko , P.‐Y. Baudin , and B. Marty , “A Steady‐State MR Fingerprinting Sequence Optimization Framework Applied to the Fast 3D Quantification of Fat Fraction and Water T1 in the Thigh Muscles,” Magnetic Resonance in Medicine 93 (2025): 2623–2639. 40033965 10.1002/mrm.30490 PMC11971504

[53] ISMRM , “Pattern Search Pulse Sequence Optimization for Cardiac MR Fingerprinting,” (2024), https://archive.ismrm.org/2024/0684.html.

