# Supplementary material for: Cardiac MR Fingerprinting at 0.55T Using a Deep Image Prior for Joint T1 , T2 , and M0 Mapping
Source: J Magn Reson Imaging. 2026 Jan 22;63(5):1365–77. doi: 10.1002/jmri.70239 (PMC13066516; doi:10.1002/jmri.70239)
Supplement: Supplementary file 1 — Data S1: jmri70239‐sup‐0001‐Supinfo.docx. [file JMRI-63-1365-s001.docx]

**Supporting materials**


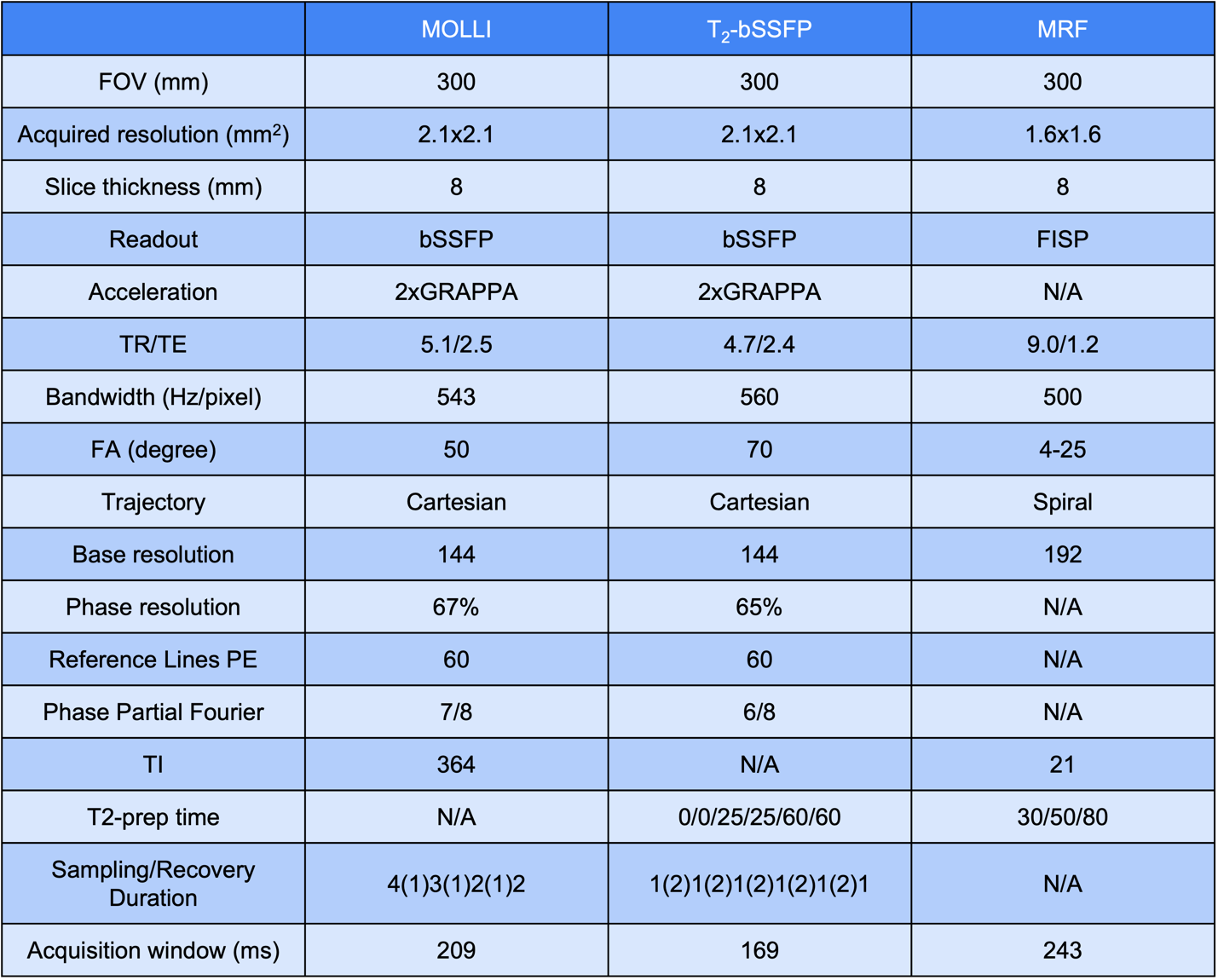


Table S1: Acquisition parameters for conventional (MOLLI and T_2_-prepared bSSFP) mapping and cardiac MRF sequences.


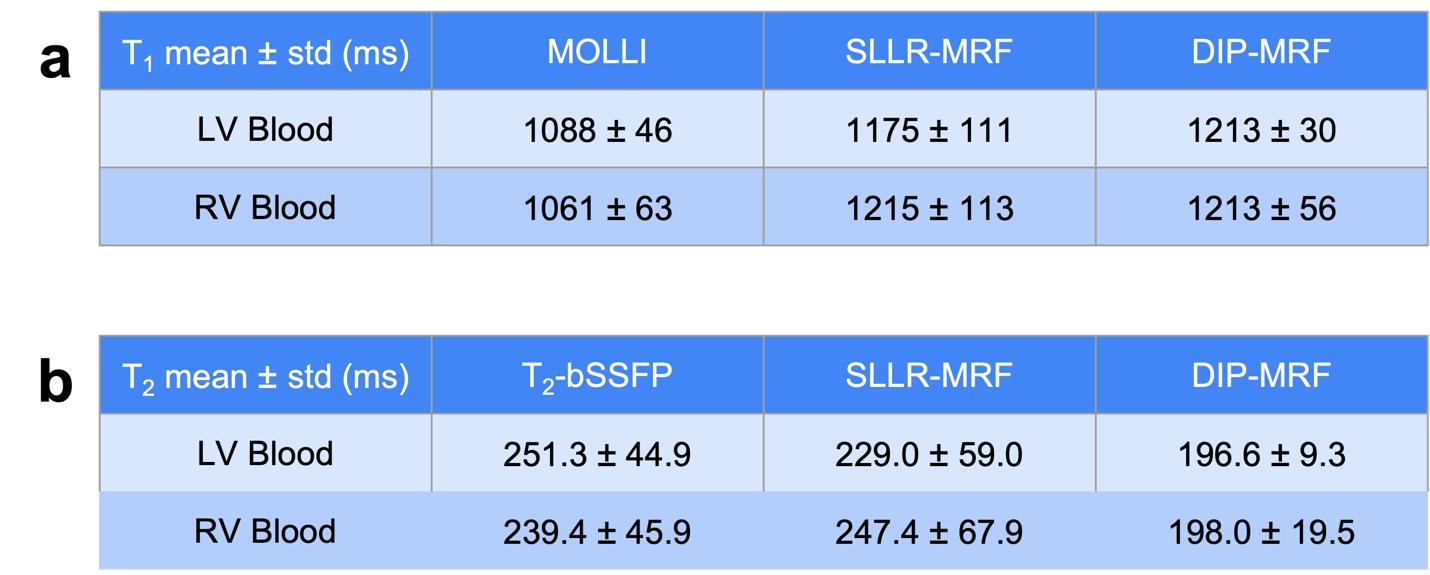


Table S2: Summary of the mean and standard deviations for (a) T1, and (b) T2 within the left and right ventricular blood pool using conventional mapping, SLLR-MRF, and DIP-MRF.


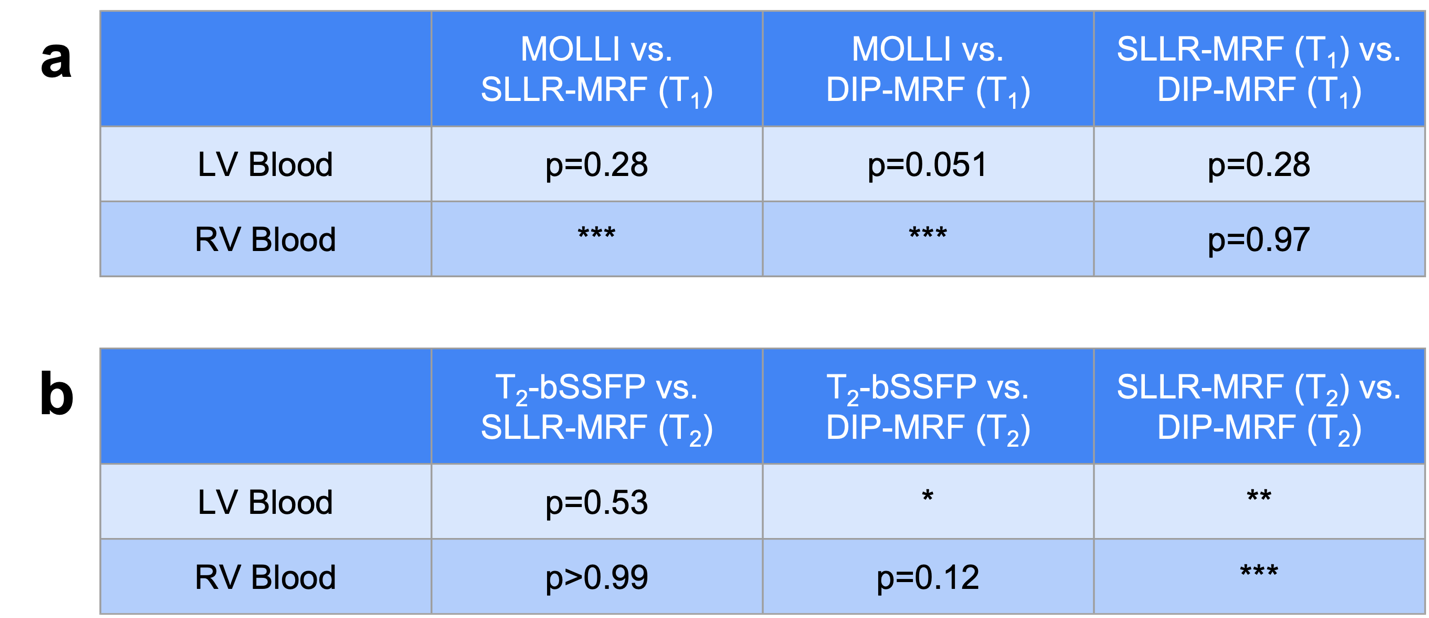


Table S3: Summary of significant differences among conventional mapping, SLLR-MRF and DIP-MRF in LV and RV blood pool values. Results are shown for (a) T_1_ and (b) T_2_. Statistically significant differences are denoted by asterisk (* p < 0.05, ** p < 0.01, and *** p < 0.001).


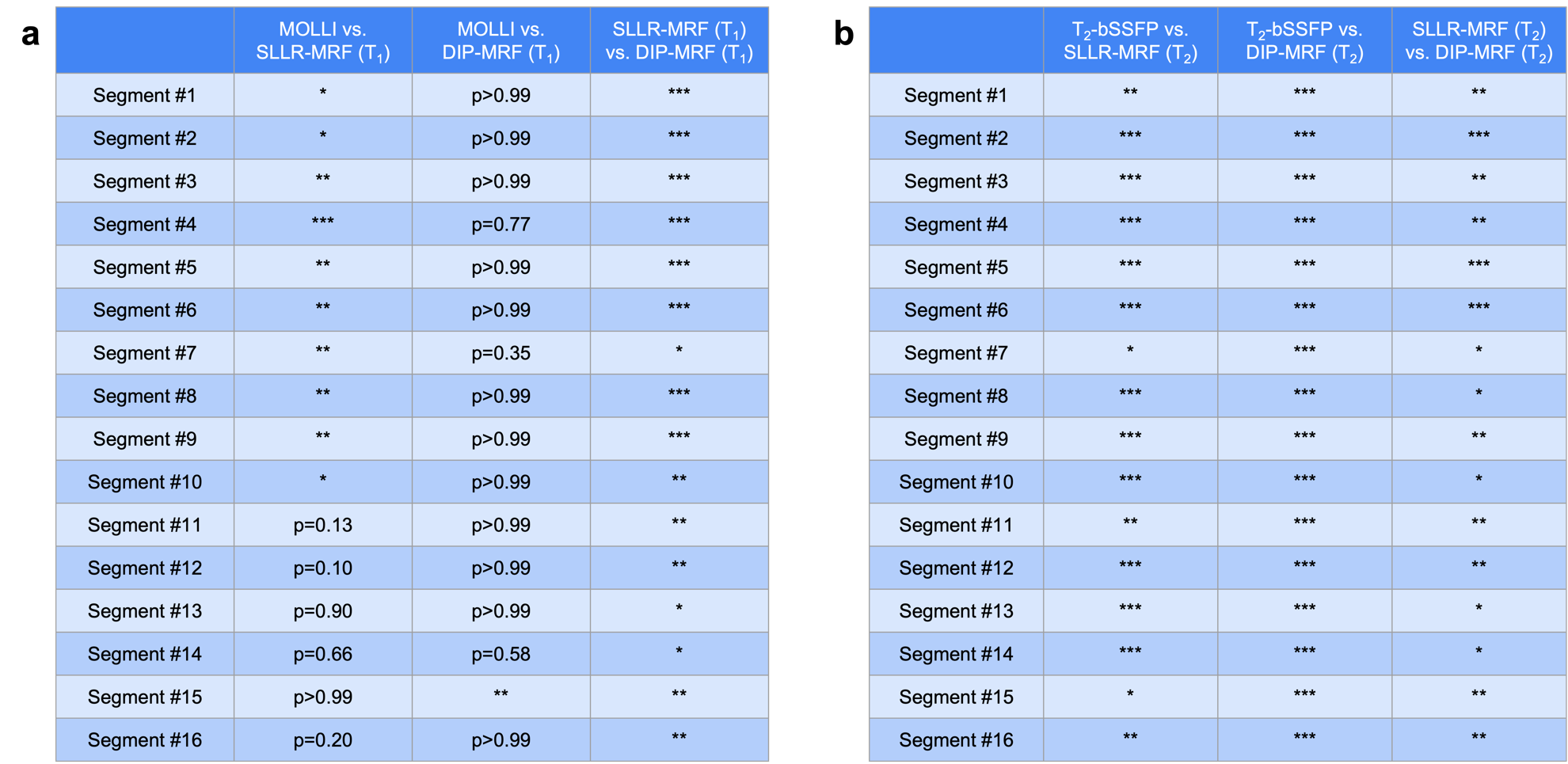
Table S4: Summary of significant differences for mean (a) T_1_, and (b) T_2_ values between conventional mapping, SLLR-MRF, and DIP-MRF within each AHA myocardial segment. Significant differences indicated by an asterisk (* p < 0.05, ** p < 0.01, and *** p < 0.001).


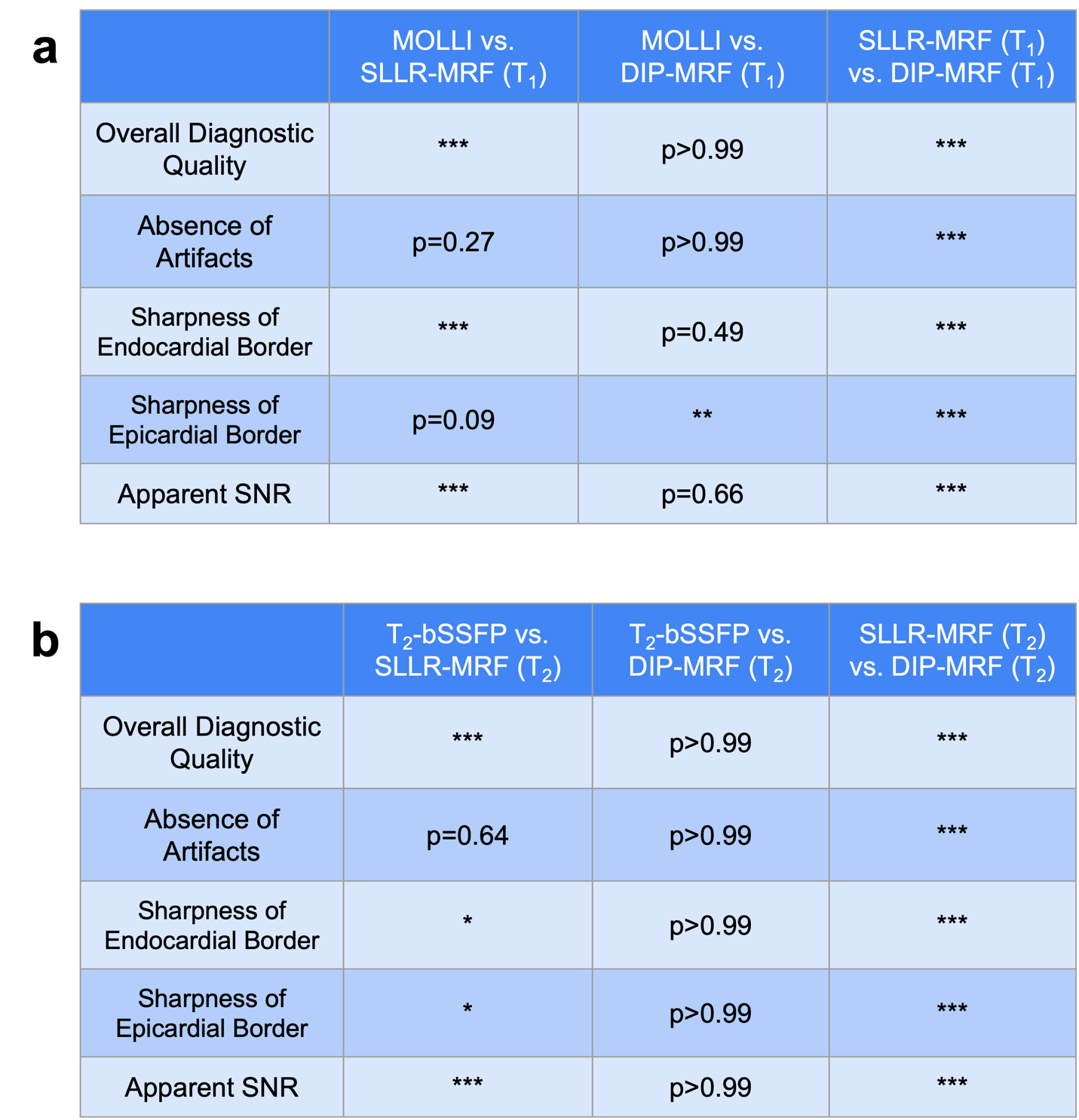


Table S5: Summary of significant differences among conventional mapping, SLLR-MRF and DIP-MRF within each category for the image rating studies. Results are shown for (a) T_1_ and (b) T_2_. Statistically significant differences are denoted by asterisk (* p < 0.05, ** p < 0.01, and *** p < 0.001).


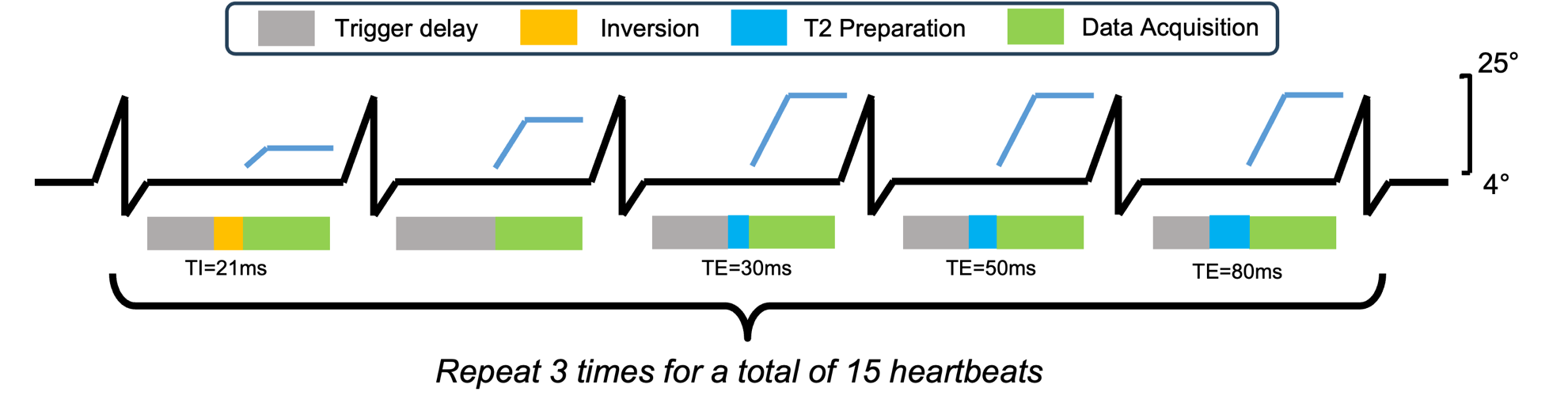


Figure S1. Schematic of the 0.55T cardiac MRF acquisition scheme. Each heartbeat includes a trigger delay followed by a diastolic data acquisition period with flip angles ranging from 4° to 25°. The first acquisition period is preceded by an inversion pulse with TI = 21 ms to enhance T_1_ sensitivity. The third, fourth, and fifth acquisition periods are preceded by T_2_-preparation modules with durations of 30, 50, and 80 ms to enhance T_2_ sensitivity. No preparation is applied during the second acquisition period. This five-heartbeat pattern is repeated three times during the 15-heartbeat scan.


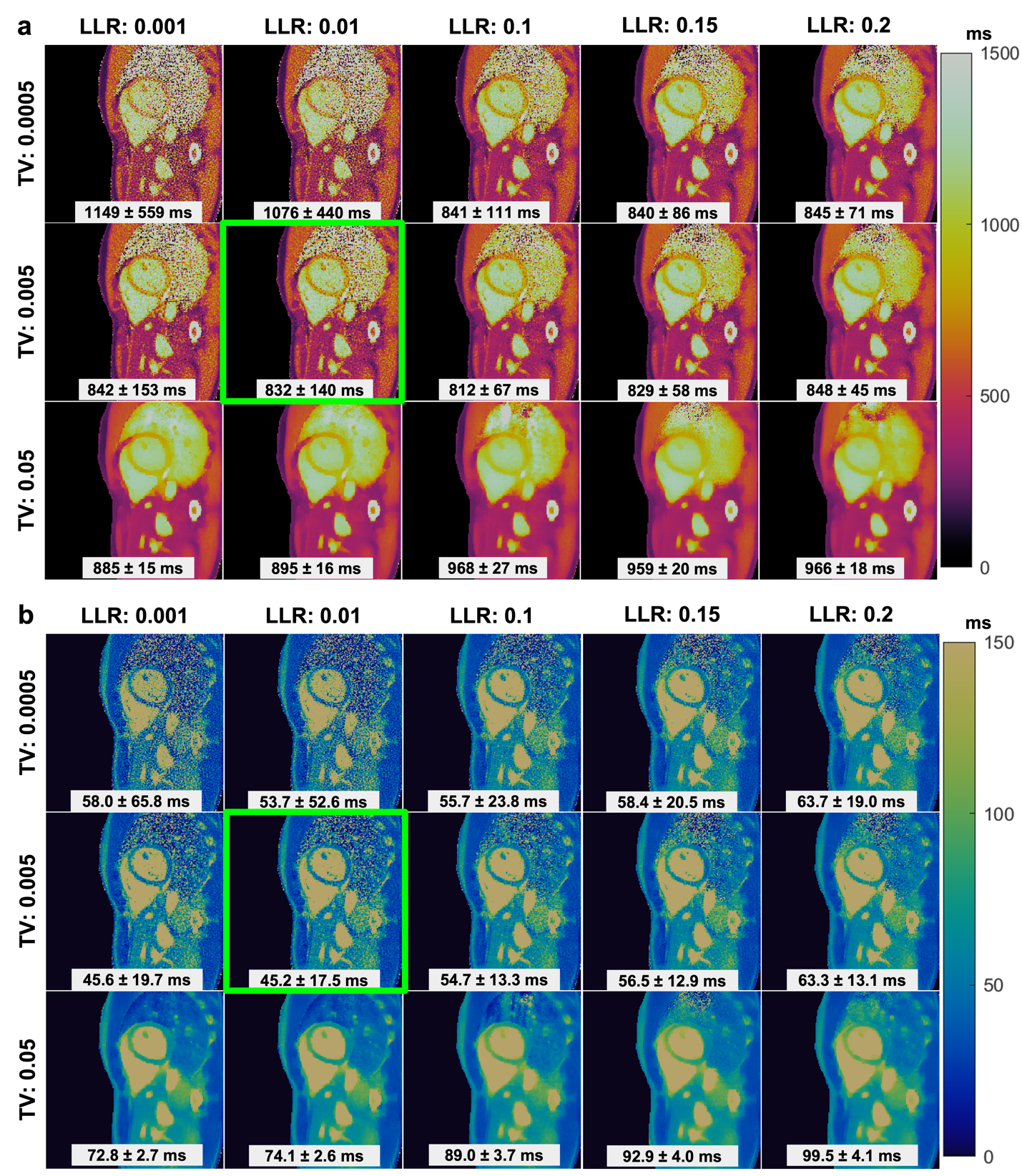


Figure S2. Tuning of SLLR-MRF regularization parameters. (a) Reconstructed T_1_ and (b) T_2_ maps are shown with varying total variation ($\lambda_{TV}$) and locally low-rank ($\lambda_{LLR}$) regularization weights in Equation 1. Columns correspond to increasing $\lambda_{LLR}$ hyperparameter values (0.001, 0.01, 0.1, 0.15, 0.2), and rows correspond to increasing $\lambda_{TV}$ hyperparameter values (0.0005, 0.005, 0.05). The regularization weights are scaled relative to the maximum signal intensity in the MRF subspace images. Stronger regularization reduces noise but leads to spatial blurring that obscures fine anatomical structures and quantitative bias. For example, increasing $\lambda_{LLR}$ beyond 0.1 (while keeping $\lambda_{TV}$ fixed) resulted in blurring between the myocardium and blood pool and an overestimation of myocardial T2 values (increasing from ~45 ms to ~ 55ms), while insufficient regularization results in noise enhancement. Empirically, values of $\lambda_{TV}$ = 0.01 and $\lambda_{LLR}$ = 0.005 (outlined in the green box) were identified as providing the best tradeoff between noise reduction and preservation of spatial resolution.


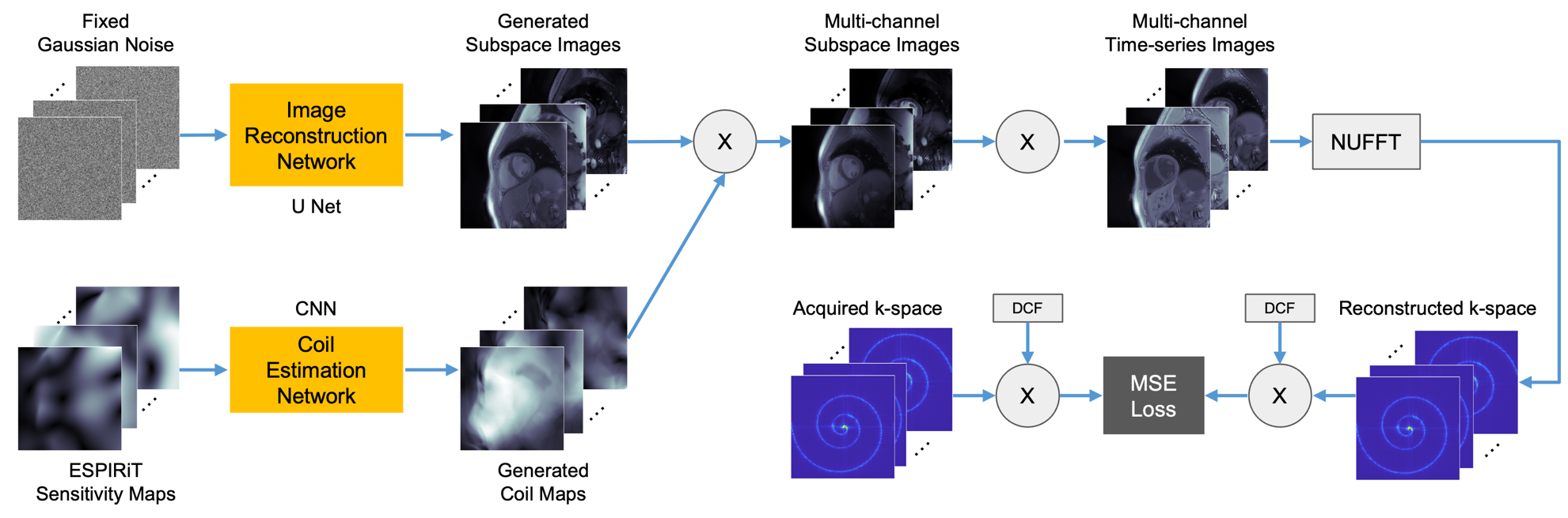


Figure S3. Schematic of the Deep Image Prior MRF (DIP-MRF) framework for reconstructing MRF subspace images and coil sensitivity maps. A fixed Gaussian noise input is passed through a U-Net, termed the Image Reconstruction Network, to generate subspace images. An initial estimate of the coil sensitivity maps obtained from ESPIRiT is passed through a convolutional neural network, termed the Coil Estimation Network (CEN), to produce a refined estimate of the sensitivity maps. The network-generated subspace images and coil maps are multiplied together to yield multi-channel subspace images. These are multiplied by $V_{k}^{*}$ to obtain uncompressed time-series images. Spiral k-space sampling is then performed using NUFFT. Both networks are jointly updated by computing the MSE loss between the reconstructed and acquired k-space data, after weighting by the spiral density compensation function.


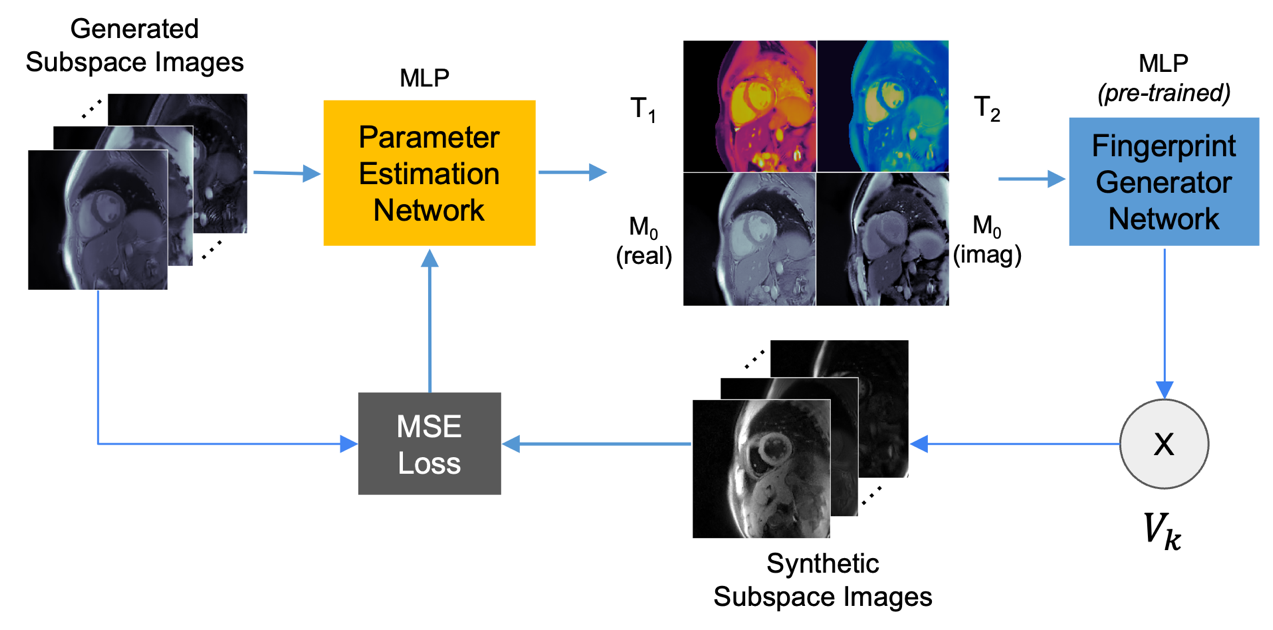


Figure S4. Schematic of the tissue property estimation portion of the DIP-MRF framework. Subspace images output by the IRN (see Figure S3) are input into a multilayer perceptron, referred to as the Parameter Estimation Network (PEN), to estimate quantitative T_1_, T_2_, and M_0_ maps. The PEN is trained in a self-supervised manner as follows. The estimated T_1_ and T_2_ maps, along with the subject’s cardiac rhythm timings (RR intervals), are passed through a second multilayer perceptron, termed the Fingerprint Generator Network, which has been pre-trained on fingerprints based on Bloch equation simulations for various T_1_ and T_2_ values and randomly simulated cardiac rhythm timings. The resulting fingerprints are then scaled by the M_0_ map to produce synthetic subspace images (by multiplication with $V_{k}$). These images are compared to those previously generated by the IRN (see Figure S3) using an MSE loss to update the PEN.


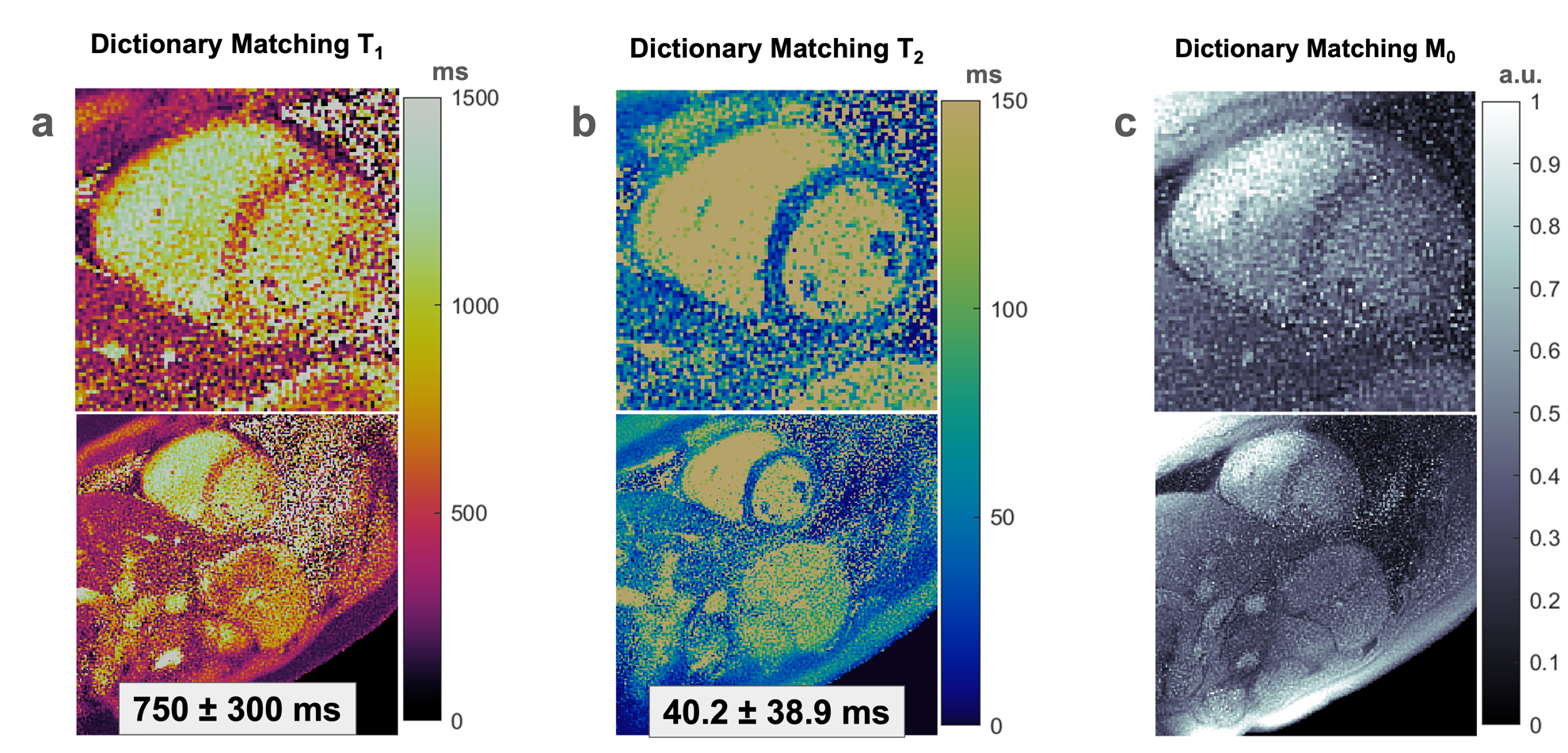


Figure S5. Examples of direct matching reconstructions for 0.55T cardiac MRF. Undersampled (zero-filled) MRF images were directly matched to the dictionary to obtain (a) T_1_, (b) T_2_, and (c) M_0_ maps. T_1_ and T_2_ values within the LV septum are reported as mean $\pm$ SD on the insets. Direct matching resulted in severe noise enhancement, necessitating the use of more advanced reconstruction methods. Reconstructions of the same dataset using SLLR-MRF and DIP-MRF are shown in Figure 2.


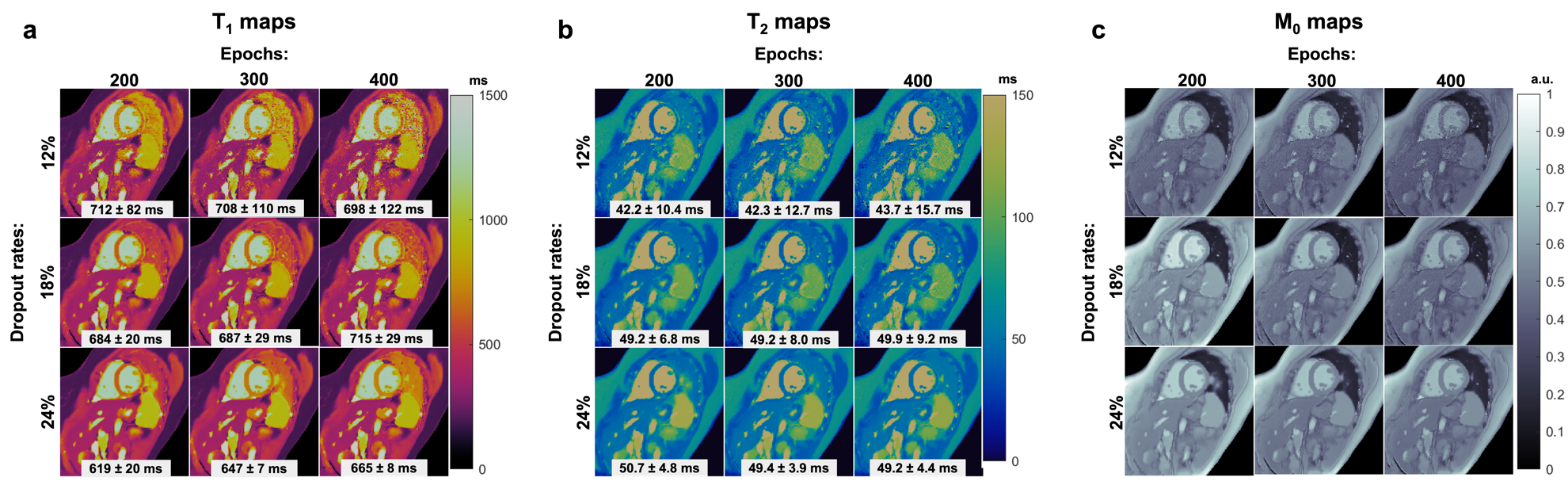


Figure S6. Full field of view maps of the same scans shown in Figure 4, illustrating the impact of dropout rate and training epochs on the reconstruction stability of DIP-MRF.
